# Fluorescent-Protein Stabilization and High-Resolution Imaging of Cleared, Intact Mouse Brains

**DOI:** 10.1371/journal.pone.0124650

**Published:** 2015-05-20

**Authors:** Martin K. Schwarz, Annemarie Scherbarth, Rolf Sprengel, Johann Engelhardt, Patrick Theer, Guenter Giese

**Affiliations:** 1 Max Planck Institute for Medical Research, Heidelberg, Germany; 2 German Cancer Research Center (DKFZ), Heidelberg, Germany; 3 Center for Molecular Biology (ZMBH), University of Heidelberg, Heidelberg, Germany; Univ Rochester Medical Ctr, UNITED STATES

## Abstract

In order to observe and quantify long-range neuronal connections in intact mouse brain by light microscopy, it is first necessary to clear the brain, thus suppressing refractive-index variations. Here we describe a method that clears the brain and preserves the signal from proteinaceous fluorophores using a pH-adjusted non-aqueous index-matching medium. Successful clearing is enabled through the use of either 1-propanol or *tert*-butanol during dehydration whilst maintaining a basic pH. We show that high-resolution fluorescence imaging of entire, structurally intact juvenile and adult mouse brains is possible at subcellular resolution, even following many months in clearing solution. We also show that axonal long-range projections that are EGFP-labelled by modified Rabies virus can be imaged throughout the brain using a purpose-built light-sheet fluorescence microscope. To demonstrate the viability of the technique, we determined a detailed map of the monosynaptic projections onto a target cell population in the lateral entorhinal cortex. This example demonstrates that our method permits the quantification of whole-brain connectivity patterns at the subcellular level in the uncut brain.

## Introduction

Visualizing connectivity between neuronal structures by light or electron microscopy almost invariably requires cutting the preparation into a series of slices. Such slices must then be individually imaged and aligned in a tedious and error-prone procedure before any three-dimensional structure can be successfully reconstructed. The need to slice the tissue results from the limited depth to which high-resolution imaging can be performed. For light microscopy, this depth limit can be greatly increased—thus removing the need to slice the tissue—by removing absorption, scattering, and optical distortion. While absorption can be reduced by draining the sample of blood and using longer wavelengths, the suppression of scattering and wave-front distortion requires the elimination of refractive-index inhomogeneity through the use of an immersion medium with a refractive index matched to the cellular components. Index-matching, also known as clearing, goes back at least a century [1] and is now commonly used when preparing biological specimens for light microscopy [2,3]. In recent years a number of protocols have been developed in which a high degree of clearing enables the entire mouse brain to be imaged at high resolution [4,5]. The almost total absence of scattering also permits the use of light-sheet microscopy, bringing with it fast imaging rates and lack of out-of-focus bleaching [6]. An apparent disadvantage is the apparent trade-off between the degree of clearing and the preservation of fluorescence from proteinaceous fluorophores (XFPs), such as EGFP (Enhanced Green Fluorescent Protein): most protocols that clear tissue effectively also result in the loss of XFP fluorescence, either immediately [4,7] or within hours or days [4,5,7–10]. Other protocols that preserve XFP fluorescence and can clear the brains of embryonic or young mice [7,9] are not effective at clearing the brains of adult mice [7,9–12]. Several recently published protocols (SCALE [9], CLARITY-based protocols [13,14] and CUBIC [15]) also preserve XFP fluorescence well and show improved whole mouse brain clearing when compared to other aqueous clearing protocols [7,10]. However, SCALE clearing takes weeks to months, depending on tissue size, and the cleared samples retain optical distortions [9]. With CLARITY-based protocols, the level of clearing and optical distortions has been reported, but not in detail [13], or refractive index mismatches remained [14]. In CUBIC-cleared brains some opacity remains detectable [15].

Imaging axonal connections throughout the structurally intact brain requires high resolution and sensitivity. Both are provided by the light-sheet fluorescence microscope (LSFM), which requires a very high level of whole-brain clearing and suitable lack of optical distortion. Here we describe and validate the FluoClearBABB protocol that allows brain-wide clearing, minimizes optical distortions and preserves the majority of EGFP and mRFP1 fluorescence. FluoClearBABB differs from other published non-aqueous protocols based on benzyl alcohol / benzyl benzoate (BABB) in that either 1-propanol or *tert*-butanol is used during dehydration and a basic pH is maintained throughout the procedure.

## Results

### Clearing parameters and EGFP fluorescence in vitro

**Fig 1:**
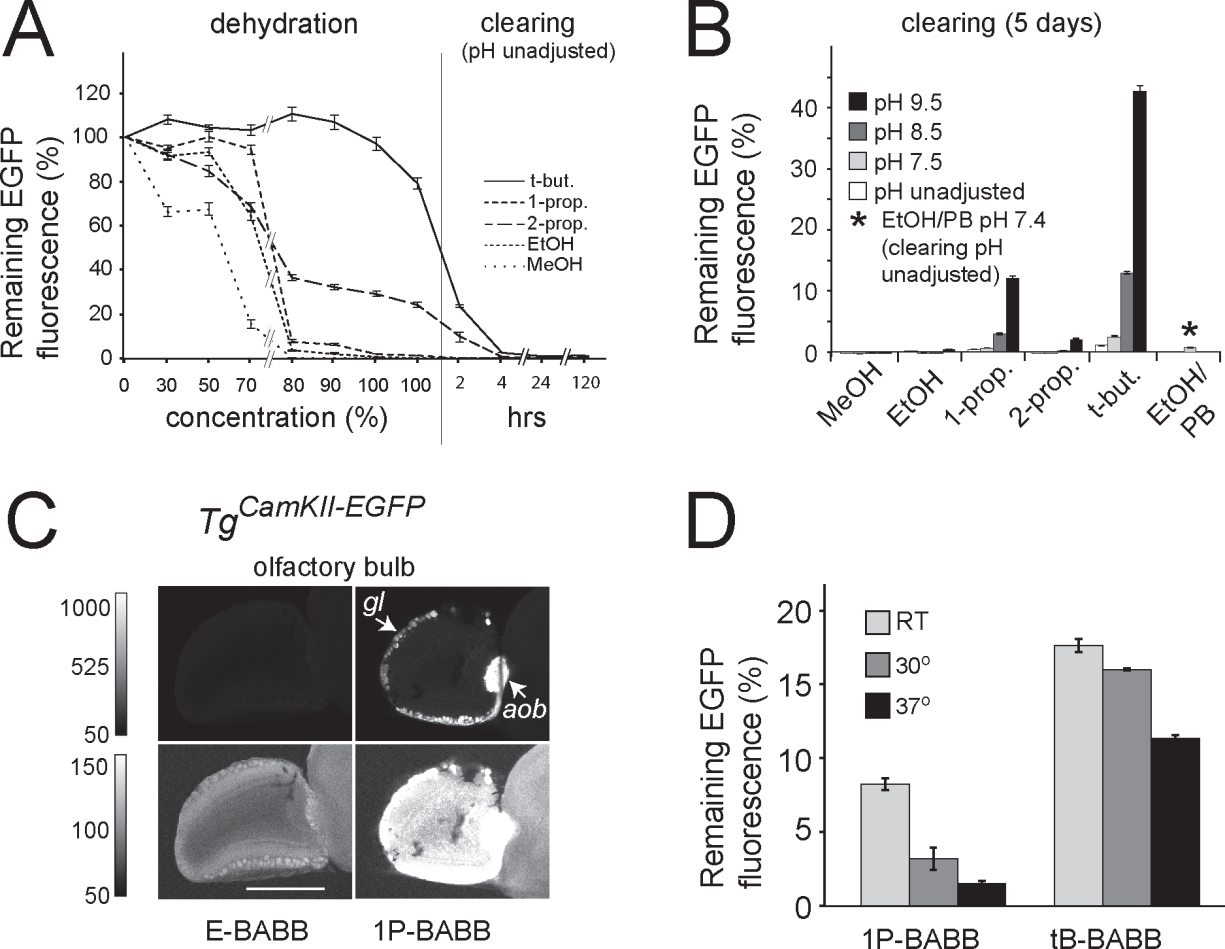
Changes in EGFP fluorescence in *E*.*coli* during dehydration and clearing. (**A**) Fluorescence of fixed, EGFP-expressing *E*. *coli* cells during dehydration and clearing. Dehydration steps: 2.5 h each (80%: 16 hours); clearing steps as indicated, all steps at RT, pH unadjusted. (**B**) Fluorescence of fixed, EGFP-expressing *E*. *coli* cells after dehydration in pH-adjusted alcohol solutions as indicated, followed by 5 d clearing at the respective pH (all steps at RT, data from different experiment). (**C**) LSFM recording (sagittal optical slice) of olfactory bulb EGFP fluorescence of a brain from a two weeks old transgenic *Tg* ^*CamKII-EGFP*^ mouse. After fixation, the two hemispheres of the brain were separated. One hemisphere was dehydrated and cleared according to Dodt et al. (“E-BABB”, left) [4], the other hemisphere using 1-propanol for dehydration and BABB for clearing, all steps at pH 9.5 (“1P-BABB”, right). For both samples, dehydration steps were 8h and 16h alternating; clearing step: 7 hours; all steps at RT. The brightness and contrast settings were set to two different linear ranges (indicated on the left) which were chosen to show the 1P-BABB sample intensity range (upper panels) and the E-BABB sample intensity range (lower panels). gl, glomerular layer; aob, accessory olfactory bulb. Scale bar, 1mm. (**D**) Fluorescence of fixed, EGFP-expressing *E*. *coli* cells after dehydration with 1-propanol, or *tert*-butanol, and additional five days in clearing solution, all steps at the indicated temperatures, all solutions at pH 9.5.

To compare fluorescence preservation for the various protocols, we used an assay based on *E*. *coli* cells expressing fluorescent protein. These cells were fixed and subsequently embedded in agarose, which allowed the quantification of fluorescence in the sample to be monitored throughout the clearing process. Using this assay we observed an almost total loss of EGFP fluorescence after dehydration (99.9% loss) when we applied the typical benzylalcohol / benzylbenzoate (BABB) clearing protocol [4]. The majority of the loss in fluorescence occurred during the dehydration steps (35.4% loss at 70% ethanol, 96.5% loss at 80% ethanol, Fig 1A). When methanol was used as the dehydrating alcohol, the loss of fluorescence was more rapid and severe (84.5% loss at 70% methanol, 99.7% at 80% methanol, Fig 1A). When the hydrocarbon content of the alcoholic component of the dehydration solution was increased, however, substantially more fluorescence was retained after dehydration (24.2% with 1-propanol, and 79.2% with *tert*-butanol (Fig 1A). In spite of this, most of the fluorescence was still lost during the BABB procedure: even when using *tert*-butanol for dehydration only 1.0% of the original fluorescence was retained after 5 days of clearing (Fig 1A).

Next, the influence of the pH during dehydration and clearing was examined. This was based on the observation of Mertz and Kim that the use of phosphate-buffered ethanolic (EtOH/PB) dehydration solutions (adjusted to pH 7.4 and with up to 96% ethanol content, leaving the pH of 100% ethanol and of the clearing solution uncontrolled) reduced the loss of EGFP fluorescence in the clearing step [11]. Using this (EtOH/PB) protocol, 21.6% of the original EGFP fluorescence remained following the last dehydration step in 100% ethanol (S1A Fig, left, EtOH/PB). However, EGFP fluorescence was barely detectable (1.6% and 1.5%) after incubation for 1 day (S1A Fig, center, EtOH/PB) or 5 days (Fig 1B, EtOH/PB), respectively, and undetectable after 16 days in a clearing solution with uncontrolled pH (S1A Fig, right, EtOH/PB). Since precipitation was observed in the clearing solution, and since phosphate buffer is a common cause of such precipitation [16], we eliminated phosphate completely and no longer buffered the solutions, instead increasing the pH to 9.5 by adding triethylamine to both the dehydration and the clearing solutions. We observed that the effect of high pH during clearing in retaining EFGP fluorescence was always positive, even if total levels varied considerably between experiments and *E*. *coli* cell preparations. This effect was most pronounced for samples dehydrated in 1-propanol or *tert*-butanol and cleared in BABB. For both 1-propanol and *tert*-butanol dehydrated samples, the fluorescence rapidly decreased when the clearing solution was at lower pH but was quite stable at high pH (1-propanol: 1 day, 0.9% at unadjusted pH, 15.4% at pH 9.5; 5 days, 0.5% at unadjusted pH, 12.1% at pH 9.5; 16 days, 0.4% at unadjusted pH, 10.5% at pH 9.5; with *tert*-butanol: 1 day, 2.5% at unadjusted pH, 46.1% at pH 9.5; 5 days, 1.1% at unadjusted pH, 42.7% at pH 9.5; 16 days, 0.7% at unadjusted pH, 44.2% at pH 9.5; Fig 1B; S1A Fig). Increasing the pH of the clearing solution to 10.5 with triethylamine did not improve fluorescence retention any further (S1B Fig). After 3 days in clearing solution at uncontrolled pH, the fluorescence could not be restored significantly by increasing the pH of the clearing solution to 9.5 for another 3 days, independent of which alcohol was used for dehydration (S1D Fig). When the samples were dehydrated with *tert*-butanol at pH 9.5, cleared with BABB pH 9.5, and rehydrated with decreasing concentrations of *tert*-butanol (pH 9.5) followed by three days in PBS, the initial fluorescence levels were maintained in the treated samples (S1D Fig), which shows that no irreversible damage to EGFP occurred under these conditions. When the pH was not controlled during dehydration with *tert*-butanol and for the first 3 h of clearing, followed by additional clearing and subsequent re-hydration with *tert*-butanol (both at pH 9.5) and 3 days in PBS, recovery remained high at 80%. It should be noted that when methanol was used instead of *tert*-butanol during dehydration only 10% of the initial fluorescence could be recovered (S1D Fig).

### Clearing and EGFP fluorescence in brains from young and adult animals

We then tested whether the dehydration and clearing parameters we had optimized *in vitro* also improve EGFP stability in optically cleared whole mouse brains. Here, we compared the levels of fluorescence preservation in the left and right olfactory bulbs (OBs) of a mouse brain (P15) expressing EGFP [17], where one hemisphere had been cleared using the original protocol (ethanol/BABB, pH unadjusted [4], which we call “E-BABB”) and the other using our 1-propanol/BABB pH 9.5 clearing protocol (“1P-BABB”). We detected only very weak fluorescence in the OB of the brain hemisphere cleared for 7 h with the E-BABB protocol, whilst we observed significant fluorescence in the OB of the hemisphere cleared with 1P-BABB (Fig 1C). After 68 days in 1P-BABB clearing solution the level of fluorescence was unchanged from its level after a mere 7 h in clearing solution (S2 Fig), indicating that the parameter values which we found to be effective *in vitro* were also highly successful in brain tissue.

**Fig 2:**
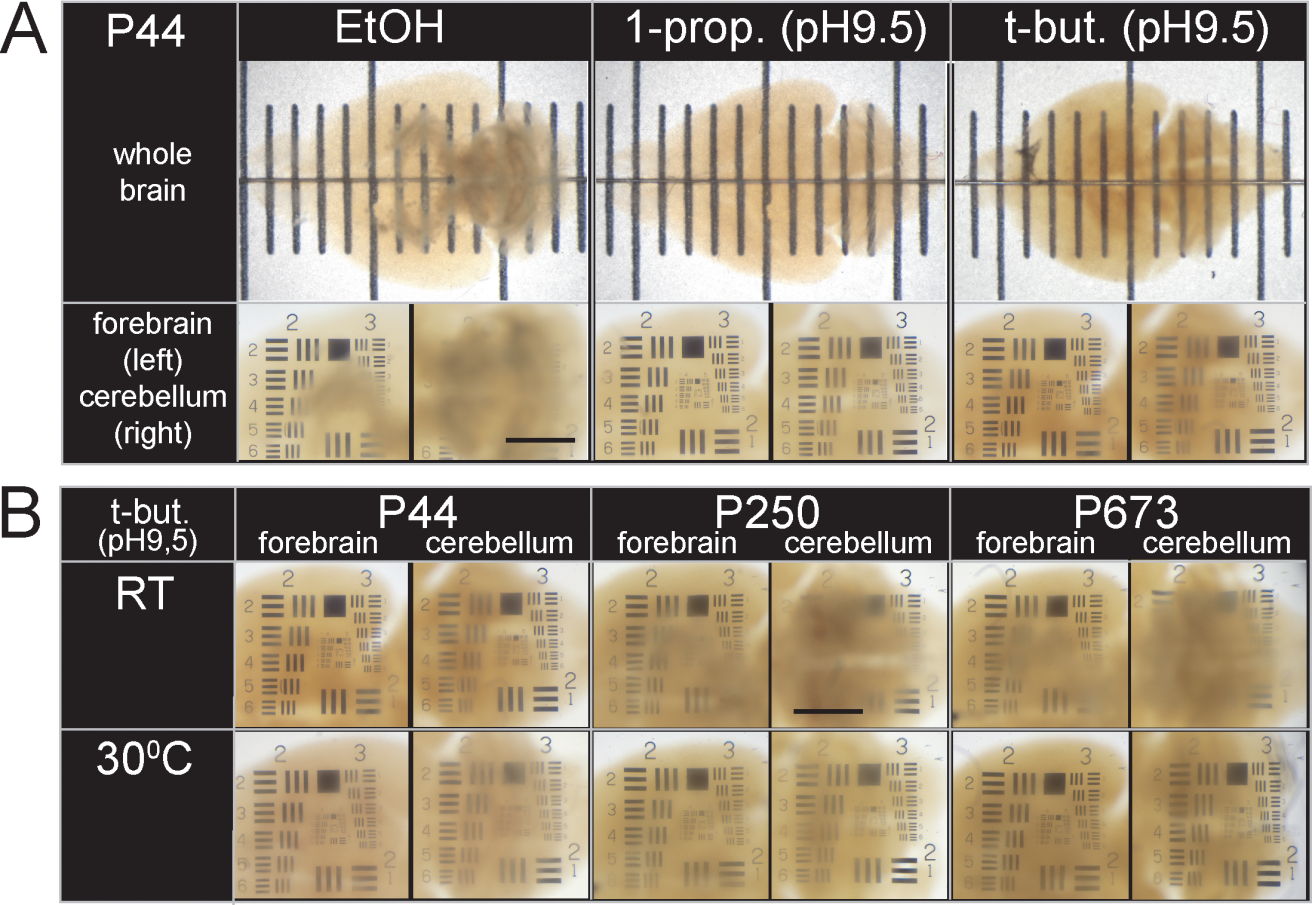
Clearing of brains using different alcohols and clearing solutions / pH adjustments. (**A**) Transmitted light images of whole mouse brains (age: P44) dehydrated with different alcohols as indicated (EtOH: pH unadjusted; other alcohols: pH adjusted to 9.5) and incubated in BABB clearing solution at the respective pH for 150 days (all incubations at RT). Brains were either placed on top of a transparent ruler (big panels; small ticks, 1 mm), or on the top of a 1951 USAF light-transmissive target (inserts; Scale bar, 1 mm). (**B**) Transmitted light images of forebrains and cerebella of adult mouse brains at different ages (P44, P250 and P673) dehydrated with *tert*-butanol (pH 9.5) and cleared with BABB pH 9.5 for 3 d; all incubations at RT (upper row) or 30°C (bottom row). P44 brains were recorded after additional 100 d storage at 4°C. The ages of the mice analyzed are indicated on the top of the image.

Using the E-BABB protocol, brains isolated from C56Bl6/N mice at postnatal day 23 (P23) were cleared well, with the exception of the cerebellum (S3A Fig). In older mice clearing was incomplete in both cerebrum and cerebellum (P50 and P67; S3B and S3A Fig). Brains from mice aged between P23 and P67 were all cleared more effectively with both variants of our new protocol than with E-BABB, in a manner more or less independent of the pH (S3A and S3B Fig **)**. In this case 1P-BABB, displaying inferior fluorescence retention *in vitro*, did provide better clearing than our *tert*-butanol / BABB pH 9.5 protocol (“tB-BABB”; Fig 2A; S3A and S3B Fig). The tB-BABB protocol left the brain darker, but this did not affect fluorescence imaging (see below). Furthermore, the 1P-BABB procedure caused less shrinkage (length changes in rostrocaudal and lateral directions were -25% and -22%, respectively) than tB-BABB (-27% / -28%), but more than the E-BABB procedures (-21% / -18%). We also tested several protocols that do not involve dehydration, but found that neither the glycerol-based variant of the CLARITY method [13] nor the urea-based SCALE methods [9] rendered mouse brains as transparent as the 1P-BABB or tB-BABB protocols did (S3C Fig). Shrinkage of brains in non-aqueous clearing solutions and the dramatic expansion of brains using the different SCALE procedures became obvious when compared to a native PFA-fixed mouse brain (S3C Fig). Clearing, shape and mechanical rigidity proved to be very stable from day 1 to 5 months with all the non-aqueous protocols tested (S4 Fig).

Exploring the temperature dependence of the clearing efficiency, we found that for older animals the clearing was more efficient at 30°C than at room temperature (RT; Fig 2B and Table 1). While *in vitro* EGFP fluorescence after tB-BABB clearing at 30°C (tB-BABB/30°C) was reduced only slightly, it dropped severely when performed at 37°C (Fig 1D). This was also true for mRFP1 (monomeric Red Fluorescent Protein 1; S1C Fig), which is frequently used as a second, spectrally distinct fluorescent marker.

**Table 1:** BABB clearing efficiency after dehydration with 1-propanol or *tert*-butanol at different temperatures.

		forebrain						cerebellum				
	Postnatal day	0–650	0–28	29–50	51–180	181–365	366–650	29–650	29–50	51–180	181–365	366–650
**1P-BABB / RT**	mean score	**3**	5	4	2.9	3	2.5	**2.2**	-	2.4	1.5	1.5
	*SD mean*.	*0*.*7*	*-*	*-*	*0*.*6*	*0*	*0*.*7*	*0*.*7*	*-*	*0*.*6*	*0*.*7*	*0*.*7*
	brains (n =)	22	1	1	16	2	2	18	-	14	2	2
**1P-BABB / 30°C**	mean score	**4.2**	-	-	4.3	4	4	**4**	-	4	4	4
	*SD mean*.	*0*.*4*	*-*	*-*	*0*.*6*	*-*	*-*	*0*	*-*	*0*	*-*	*-*
	brains (n =)	5	-	-	3	1	1	5	-	3	1	1
**tB-BABB / RT**	mean score	**3.5**	4	3	3.5	3.5	3.5	**3.2**	-	3.6	2.5	3
	*SD mean*.	*0*.*5*	*-*	*-*	*0*.*5*	*0*.*7*	*0*.*7*	*0*.*8*	*-*	*0*.*5*	*0*.*7*	*1*.*4*
	brains (n =)	14	1	1	8	2	2	9	-	5	2	2
**tB-BABB / 30°C**	mean score	**3.8**	-	3.9	3.7	3	3	**3.2**	3	3.3	4	4
	*SD mean*.	*0*.*5*	*-*	*0*.*5*	*0*.*6*	*-*	*-*	*0*.*7*	*0*.*6*	*0*.*7*	*-*	*-*
	brains (n =)	56	-	27	27	1	1	56	27	27	1	1

All steps were performed either at room temperature (RT) or at 30°C as indicated, and at pH 9.5. The transparency scores of the forebrain and the cerebellum of each brain were evaluated on an USAF Wheel Target (cf. Fig 2) by an investigator blind to the procedure. Score 0 = totally uncleared; 1 = most areas uncleared; 2 = large uncleared areas and / or strongly refractive areas; 3 = some uncleared and / or some moderately refractive areas; 4 = few slightly uncleared areas and / or some weakly refractive areas; 5 = clear, no visible refraction.

Clearing brains with the tB-BABB/30°C procedure emerged as the most reliable protocol, in particular for fluorescence imaging of brains from adult mice, as it provides near optimal fluorescence preservation in combination with excellent clearing. The other variants of our new protocol described here may prove advantageous if fluorescence preservation or superior clearing are important. “FluoClearBABB” will now be used as an umbrella term encompassing all variants of our clearing protocol.

### Whole-brain imaging with light-sheet microscopy

**Fig 3:**
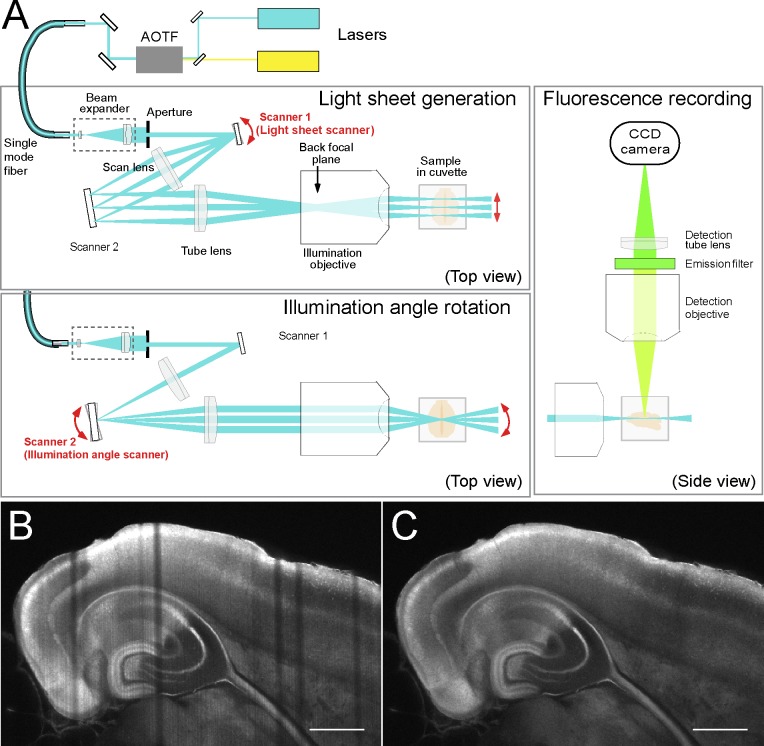
Light-sheet microscope (LSFM) setup and shadow suppression. (**A**) Beam path for light sheet-generation / fluorescence excitation (blue: left panels, top views) and for fluorescence excitation (blue) and detection (green) (right panel, side view). Scanner 1 generates the light sheet, while scanner 2 controls the illumination angle rotation. During recording, both scanners are active simultaneously. (**B, C**) Suppression of shadow stripes by scanner 2. Single xy-plane Venus FP fluorescence images from a fluorescent mouse brain prepared from a *Tg* ^*Y5RitTA⁄Y1RVenus*^ [39] mouse at P13, cleared with the 1P-BABB method and recorded with our LSFM (excitation. 514 nm; emission, bandpass 520–550 nm) demonstrate the effect of scanner 2 that rotates the light sheet (for technical details, see Fig 3A). (B) scanner 1 ON, scanner 2 OFF; (C) both scanners ON. Illumination from lateral side of left hemisphere (from top of panels), imaging from dorsal (from viewer’s direction). Scale bar, 1 mm.

Imaging a whole mouse brain at an xyz resolution of around 1.6 * 1.6 * 3.2 μm/voxel and a pixel dwell time of 2 microseconds requires about 24 h using a single-point scanning microscope. We therefore built a light-sheet fluorescence microscope (LSFM), which provides higher speed recordings (about 4 h for a whole brain at this resolution) together with greatly reduced out-of-focus bleaching. The LSFM, unlike a standard confocal or two-photon microscope, requires the samples to be largely free of scattering, which can be achieved by clearing. However, even with cleared samples LSFM measurements generate shadows due to residual refractive index inhomogeneity, in particular when using a stationary light-sheet generated with a cylindrical lens [18]. This effect becomes more severe with increasing sample size, but can be counteracted by periodically rotating the light-sheet around the center of the field of view [18]. Sweeping a double cone-shaped beam quickly across the field of view to generate the light sheet [19] also reduces shadow formation when compared to cylinder lens-based light-sheet generation. We combined the two approaches, which required the use of 2 scanners but eliminated shadow generation almost completely (Fig 3A–3C). The use of highly corrected objectives—which is possible because a cylindrical lens is no longer needed–, whilst coupling the excitation light through an optical fiber, ensured the precise overlap of light sheets of different wavelengths.

Low-resolution stacks (7.7 * 7.7 * 7.7 μm voxel size) from a cleared, intact adult mouse brain can be acquired in about 10 minutes of recording time per channel (with no or minimal tiling, depending on sample size) by using objectives with low magnification and low numerical aperture (NA) but with a large field of view in both the excitation and imaging paths. Note that, unlike in a confocal microscope where the resolution along the z axis falls with the square of the NA of the imaging objective, in the LSFM the z resolution declines only linearly with the NA of the illumination objective. For higher resolution, we recorded mosaics of z stacks for each hemisphere using objectives with higher NA and magnification.

**Fig 4:**
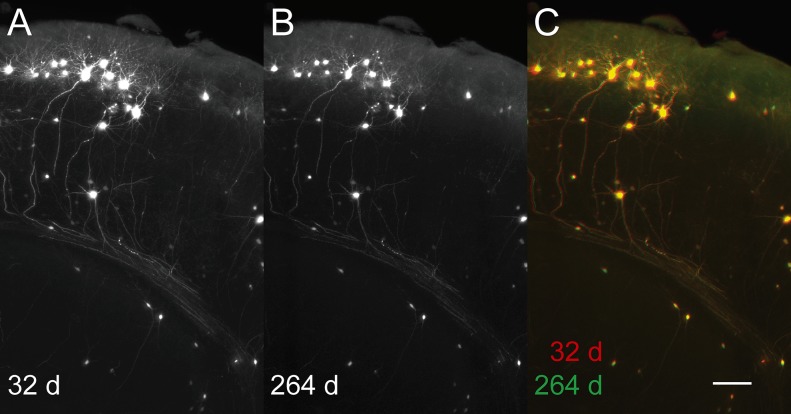
Long term preservation of fluorescence and sample geometry. Light sheet microscope horizontal recordings of green fluorescence (RABV*ΔG-EGFP*) from a p70 mouse brain were taken at day 32 and day 264, respectively, after the onset of clearing. Corresponding subvolumes of the entorhinal cortex / hippocampus region were selected with ImageJ and upscaled to a cubic voxelsize of 1.61 microns with AMIRA software. The day 264 dataset was 3D aligned to the day 32 dataset by affine transformation with AMIRA (Lanczos interpolation). Since the day 32 dataset images had been recorded at three times the excitation power (4.65 microjoule per mm light sheet width) compared with the day 264 dataset images (1.55 microjoule per mm light sheet width), we accounted for this difference by adjusting the image display range with ImageJ accordingly (Set Display Range (32d): 70–1269; (264d): 63–462; with the lower value of each range representing the image background outside the brain tissue signal area). All other recording and image analysis parameters were maintained. Image shows overlapping z maximum projections (50 microns z projection size) of day 32 (**A**), day 264 (**B**) and an overlay of day 32 (red) and day 264 (green) EGFP fluorescence (**C**). Bar, 100 microns.

Fluorescence intensity and sample shape were very stable: one brain, having been stored at 4°C after tB-BABB/30°C clearing, was imaged completely after ~ 1 month in clearing solution and re-imaged ~ 8 months later: corresponding subvolumes from the entorhinal cortex / hippocampal region showed only a minor loss of signal intensity over time (Fig 4A and 4B; S1A and S1B Video) and an almost perfect match when aligned by affine transformation including adjustment in scale by < 1% (Fig 4C; S1C Video). In both recordings, the cellular morphology, as revealed by EGFP fluorescence—including dendrites and axons—, was well preserved (Fig 4A–4C; S1 Video). Even the acquisition of multiple high-resolution stacks from the same sample region did not cause significant photobleaching (data not shown).

### Imaging neurons and their monosynaptic projections to the entorhinal cortex

To visualize long-range axonal projections, we injected a cocktail of a modified rabies virus and two recombinant adeno-associated viruses into the entorhinal cortex (EC) of a P70 mouse [20,21]. We chose the EC for stereotaxic virus delivery because it receives long-range projections from different, widely distributed brain regions, including but not limited to the olfactory bulb (OB), the piriform cortex (PiC) and a number of subcortical nuclei [22,23]. The modified rabies virus (RABV*∆G-EGFP*(EnvA)) was envelope A (EnvA)-pseudotyped, glycoprotein-deficient and expressed EGFP. The two recombinant adeno-associated viruses expressed the TVA receptor (rAAV-mRFP1-IRES-TVA virus) and the rabies virus glycoprotein (RG) (rAAV-RG virus), respectively. After stereotaxic injection of the virus cocktail, the modified rabies virus selectively infected those neurons expressing the TVA receptor. The rAAV driven expression of RG in TVA expressing cells allowed subsequent transsynaptic retrograde transport of the modified rabies virus to monosynaptically connected cells. Note that after the injection of the virus cocktail into the EC, most of the mRFP1- and EGFP-expressing cells also expressed RG: firstly because the spread/diffusion of the rabies particles is less than that of the rAAV particles (RABV, ~200 nm; rAAV, ~20 nm); and secondly, due to the equimolar mixture of both rAAV viruses (~ 10^12^ rAAV particles /ml) [21,24].

**Fig 5:**
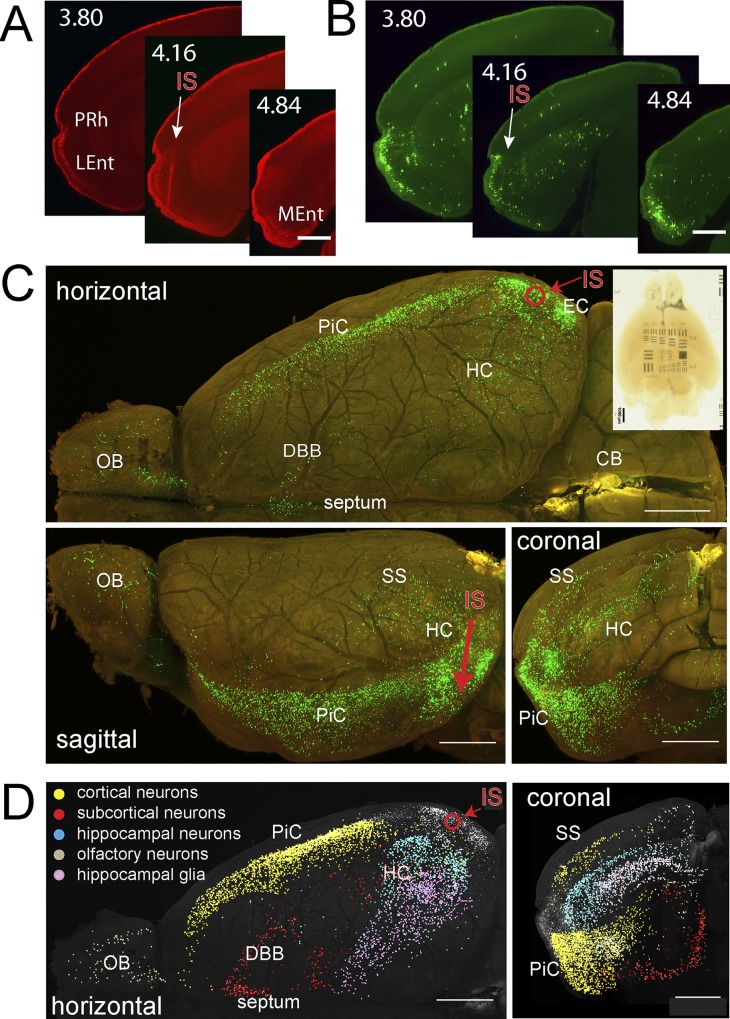
Visualization of neurons monosynaptically connected to RABV*ΔG-EGFP*(EnvA) infected neurons in EC. The fixed brain was cleared with tB-BABB/30°C for 64d. Red fluorescence (rAAV- *mRFP1-IRES-TVA*) and green fluorescence (RABV*ΔG-EGFP*) were recorded with the LSFM(xy pixel size, 1.61 μm; z-step size, 3.23 μm). Maximum projections were generated after correction for tissue-generated signal attenuation. (**A, B**) Maximum projections (50 μm) from coronal re-slices of the horizontal recording of red fluorescence (A, rAAV- *mRFP1-IRES-TVA*) and green fluorescence (B, RABV*ΔG-EGFP*). The precise position of each projection is given as distance to bregma according to [44]. Injection site and direction are indicated by a white arrow. Prh, Perirhinal Cortex; LEnt, Lateral Entorhinal Cortex; MEnt, Medial Entorhinal Cortex; IS, injection site. Scale bar, 0.5 mm. (**C**) Red/green overlays of maximum intensity projections generated from z stacks of stitched xy horizontal mosaic planes (top), and from sagittal (bottom left) and coronal re-slices (bottom right) of these z stacks. Bright green structures show RABV*ΔG-EGFP*-positive cells. The red circle and the red arrow indicate the positions of the dorsoventral virus injection channel in horizontal and sagittal views, respectively. The transmission image of the whole cleared brain located on top of a translucent USAF 1951 resolution target is shown in the upper right corner. Scale bars, 0.5 mm. (**D**) Maximum projections (left, horizontal; right, coronal) of a KNOSSOS 3D skeleton dataset generated from the green channel dataset. Each dot indicates the position of an EGFP-labeled, manually marked cell. Color coding: grey, cells in injection region (EC area); marked cells located outside the injection area are color coded as indicated. Respective greyscale maximum projections of the green channel fluorescence were overlaid to depict tissue location. OB, Olfactory bulb; DBB, Diagonal Band of Broca; PiC, Piriform Cortex; SS, Somatosensory Cortex; HC, Hippocampus; EC, Entorhinal Cortex; IS, injection site. Scale bars, 0.5 mm.

**Fig 6:**
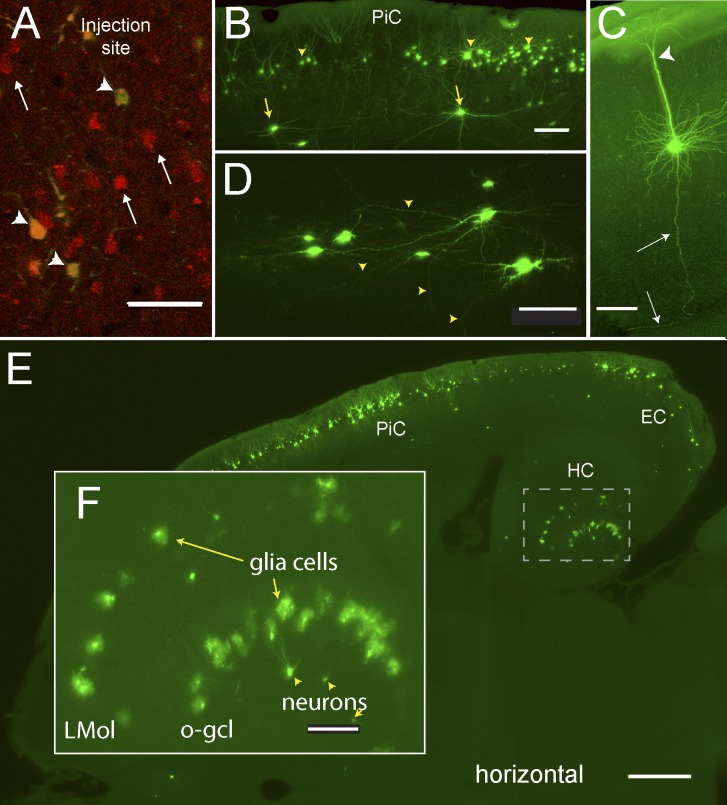
Visualization of cell bodies and neurites in a tB-BABB cleared mouse brain. Brain sample same as Fig 4; original dataset (LSFM recording, horizontal): same as Fig 4, except for (A). (**A**) Confocal recording (single plane) from EC layer 3 near the injection site taken after 271 days in clearing solution. Overlay of red channel (rAAV-driven *mRFP1* fluorescence, arrows) and green channel (RABV*ΔG-EGFP* fluorescence). Double-infected cells expressing rAAV-driven *mRFP1* plus RABV*ΔG-EGFP* fluorescence are indicated by orange to green color (arrowheads). Recording direction: from brain surface (positioned perpendicular to optical axis) to interior. Scale bar, 100 μm. (**B**) LSFM 50 μm z range maximum projection from a subvolume of the PiC area (RABV*ΔG-EGFP* fluorescence). Arrowheads indicate some Layer 2 neurons, arrows some interneurons. Scale bar, 100 μm. (**C**) LSFM 50 μm z range maximum z projection from a subvolume of the auditory cortex region (RABV*ΔG-EGFP* fluorescence) showing a cortical layer 5 cell with a big apical dendrite (arrowheads), basal dendrites at the soma, and a basal axon (long arrows). Scale bar, 100 μm. (**D)** LSFM 90 μm z range maximum projection from a subvolume of the septal area (RABV*ΔG-EGFP* fluorescence) 2.3 mm below brain surface revealing neurite structures (arrowheads). Scale bar, 100 μm. (**E**) LSFM 20 μm z range maximum projection (RABV*ΔG-EGFP* fluorescence) from a subvolume of the PiC/EC/HC region, showing neurons from cortical EC and the PiC regions and glia from the HC region projecting to the injected EC neurons. Scale bar, 500 μm. (**F**) Hippocampal DG area enlarged from (E), showing glia cells in the outer granule cell layer (o-gcl) and stratum lacunosum moleculare (LMol), and neurons in the DG hilus region (arrowheads). PiC, Piriform cortex; EC, Entorhinal cortex. Scale bar, 100 μm.

The injection channel could be identified in the LSFM horizontal recordings and in coronal re-slices of them by the presence of mRFP1 fluorescence particle clusters (Fig 5A; S2 Video). These most likely represent leftover cellular debris from early rAAV-infected cells that had died within the first 10 days after the virus cocktail injection. The injection channel runs from the cortical surface through the Perirhinal Cortex (PRh) and layers 2/3 of the caudal lateral EC (LEnt) (Fig 5A). In neurons, the strongest mRFP1 fluorescence was detected in layer 2 cell bodies of a rostral subfield of the lateral EC, in the upper half of layer 2a extending into the adjacent PRh region, and more caudally in layer 2 of the medial EC (MEnt) (Fig 5A). These rAAV-positive regions also contain EGFP positive cells, which represent directly or trans-synaptically RABV*∆G-EGFP* transduced cells (Figs 5B and 6A; S2 Video and S3 Video). Outside the EC region, trans-synaptic spreading of RABV*∆G-EGFP* could be recognized from EGFP-positive cells in various regions of the cerebrum (Fig 5C; S3 Video). As expected, the vast majority of such cells were found in the cerebral cortex and in hippocampal subfields of the ipsilateral hemisphere (Fig 5C and Table 2; S3 Video)[22].

**Table 2:** EGFP positive cell counts in the 3D skeleton dataset generated with KNOSSOS.

	Injection site		Ipsilateral (without injection site)		Contralateral	
	Neurons	Glia	Neurons	Glia	Neurons	Glia
**Total cells**	**2319**	**32**	**3683**	**903**	**85**	**0**
**Cortical cells**	**2319**	**32**	**2385**	**17**	**58**	**0**
**Subcortical cells**			**325**	**5**	**22**	**0**
**Hippocampal cells**			**863**	**880**	**5**	**0**
**Olfactory cells**			**110**	**1**	**0**	**0**
**Cortical brain regions**						
Cortex			235	0	13	0
Piriform Cortex			2093	17	34	0
Ectorhinal Cortex	41	0			0	0
Perirhinal Cortex	101	1			1	0
Lateral Entorhinal Cortex	1536	3			6	0
Medial Entorhinal Cortex	641	28			4	0
Agranular Insular Cortex			1	0	0	0
Dorsal Endopiriform Nucleus			42	0	0	0
Olfactory Nucleus			8	0	0	0
Ventral Orbital Cortex			4	0	0	0
Dorsal Peduncular Cortex			2	0	0	0
**Subcortical regions**						
MCPO			8	0	0	0
Thalamic Nuclei			60	0	0	0
Lateral Hypothalamic Nuclei			7	0	0	0
Medial Septum			68	0	21	0
HDB			65	0	0	0
VDB			42	0	0	0
Nucleus Accumbens			0	1	0	0
Amygdaloid Nuclei			74	2	0	0
Striatum			1	2	0	0
Corpus Callosum			0	0	1	0
**Hippocampus**						
Subiculum			204	80	1	0
Hippocampal alveus			0	27	0	0
External Capsule			103	8	0	0
CA1 Stratum pyramidale			191	0	0	0
CA1 Stratum oriens			42	0	0	0
CA1 Stratum radiatum			62	0	0	0
CA3 Stratum pyramidale			131	0	0	0
CA3 Stratum oriens			20	0	3	0
CA3 Stratum radiatum			54	0	0	0
(DG) Dentate Gyrus			30	487	1	0
Stratum lacunosum moleculare			26	278	0	0
**Olfactory bulb**						
Glomerular Layer			1	0	0	0
Mitral Cell Layer			109	1	0	0

The LSFM recorded dataset of the virus injected mouse brain shown in Fig 5 was analyzed in KNOSSOS after manual classification of the EGFP positive cells using the Allen Mouse Brain Atlas and Brain Explorer2 software. The number of EGFP positive cells and their distribution in different brain regions are given for the virus injected ipsilateral and the contralateral site of the brain. EGFP positive cells in the ipsilateral site, outside the injection region, and EGFP positive cells from the contralateral site are monosynaptically connected to RABV*∆G-EGFP* producing neurons in the ipsilateral hemisphere. Irrelevant cells were left empty.

The high image quality of the LSFM recordings allowed us, using KNOSSOS software [25], to manually assign the EGFP-positive cells to individual brain regions, thus generating a 3D map of cell populations monosynaptically connected to neurons of the virus cocktail-injected EC region and visualized by RABV*∆G-EGFP* retrograde labelling (Fig 5D; S4 Video). Our analysis revealed monosynaptic innervation of the EC from Piriform Cortex (PiC), olfactory bulb (OB) mitral cells, neurons in the dorsal and lateral olfactory nucleus, the medial septum (MS) including the ventral diagonal band (VDB), the horizontal diagonal band (HDB), the magnocellular preoptic nucleus (MCPO), several thalamic nuclei, the lateral hypothalamic nuclei, the Ectorhinal cortex (ECT), the perirhinal cortex (PRh), different cortical and hippocampal regions, and the subiculum (Table 2; S4 Video).

In summary, we found RABV*∆G-EGFP* expressing neurons in ipsilateral cortical (2385 neurons), subcortical (325 neurons) hippocampal (863 neurons) and olfactory (110 neurons) brain regions (Table 2). Very few neurons in contralateral cortical, subcortical and hippocampal brain regions could be detected (58, 22, and 5 neurons, respectively) indicating that contralateral neurons provide less than 3% of the input into the injected EC region (Table 2). In addition, the high-resolution images revealed that, in the ipsilateral hemisphere, 50.5% (880 cells) of the EGFP labelled cells in the hippocampus, but only 0.8% (23 cells) outside the hippocampus displayed a glia-like morphology (Fig 6E–6F; S5 Video). Glia cells were most abundant in the molecular layer of the DG and in the stratum lacunosum moleculare (Table 2).

**Fig 7:**
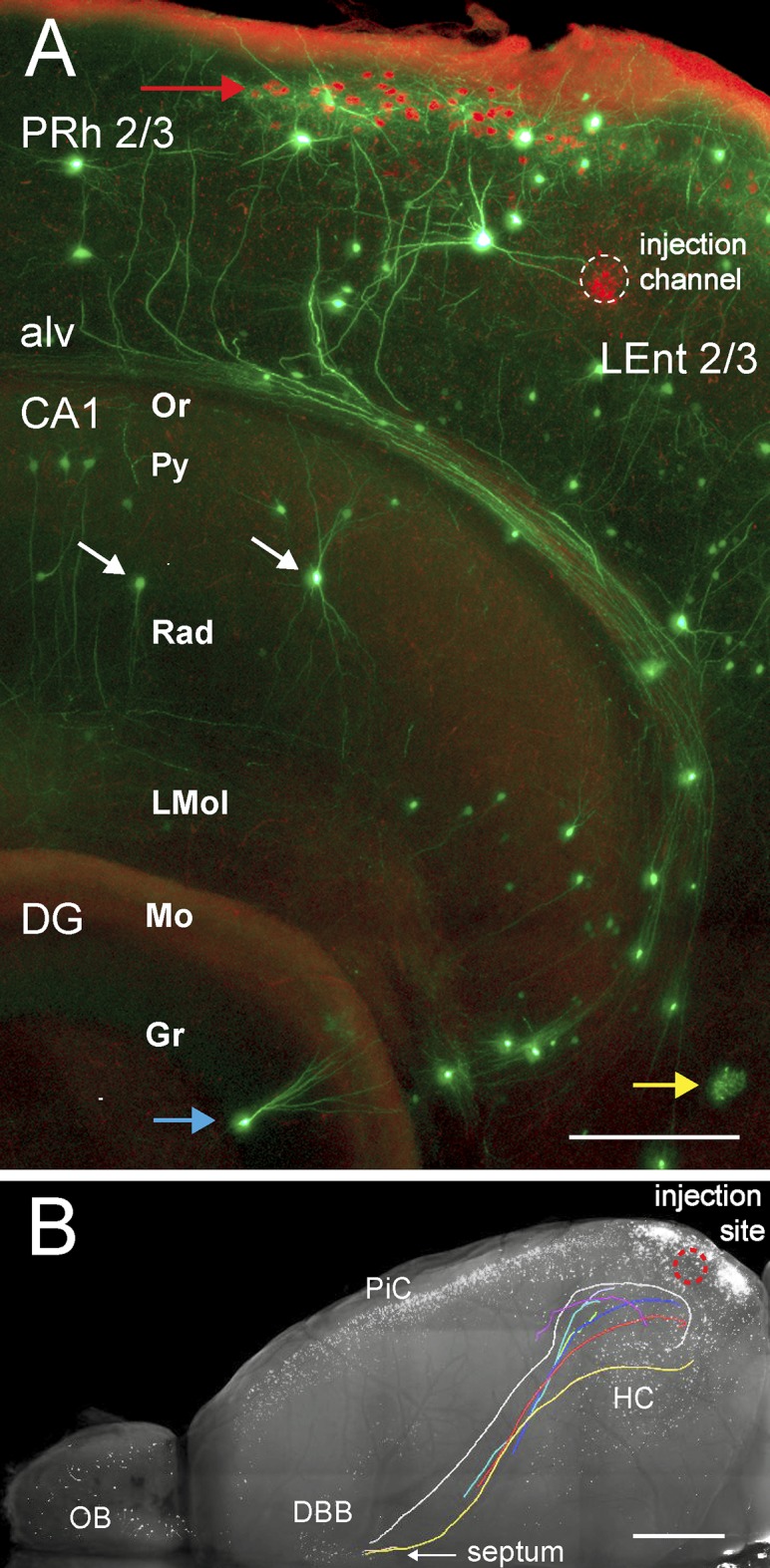
Neuronal cells and neurites in the ipsilateral injected EC/hippocampal area. Brain sample same as Fig 4; original dataset (LSFM recording, horizontal): same as Fig 4. (**A**) Green/red overlay of LSFM 50 μm z range maximum projections from a subvolume of the EC/HC area. Green channel: RABV*ΔG-EGFP* fluorescence revealing different cell types with dendrites and axon bundles. Red channel, rAAV-labeled cells (red arrow) below the brain periphery, which shows red autofluorescence. Dotted circle encloses the red fluorescence of the injection channel, which runs almost perpendicular to the image plane. White arrows, interneurons; blue arrow, granule cell; yellow arrow, glia cell. PRh 2/3, layers 2/3 of perirhinal cortex; alv, alveus of the hippocampus; CA1, Ammons Horn field CA1; Or, oriens layer; Py, pyramidal cell layer; Rad, radiatum layer; LMol, lacunosum moleculare; DG, dentate gyrus; Mo, molecular layer, Gr, granular layer; LEnt 2/3, layers 2/3 of lateral entorhinal cortex. Colors representing fluorescence signals were partially desaturated with Adobe Photoshop. Scale bar, 200 μm. (**B**) Long-range axonal projections traced in 3D with KNOSSOS and overlaid onto a green channel maximum z projection rendered as greyscale (cf. Fig 4C, top panel). Colored axon trajectories were generated with Amira from 3D KNOSSOS datasets. Arrow points to septum. Scale bar, 1 mm.

The same dataset displayed well-resolved dendrites and axons of cells located not only near the sample surface (Fig 6B and 6C), but also several millimeters deep (Fig 6D). It revealed trajectories of axons from neurons projecting onto triple infected EC cells (Fig 7A), and some axons could be manually traced from the hippocampal region along the entire rosto-caudal as well as dorso-ventral extension of the cerebrum using KNOSSOS software (Fig 7B; S6 Video). This is a clear demonstration of the sensitivity and in-depth resolution performance of our LSFM imaging system in combination with whole brain clearing with the FluoClearBABB method.

## Discussion

### Clearing and EGFP fluorescence

In this study we demonstrate that careful selection of the alcohol used during dehydration and maintenance of basic pH during clearing allows effective clearing of the brains of adult and aged mice while preserving a large fraction of the EGFP and mRFP1 fluorescence. Clearing is sufficient for the recording of high-resolution image stacks with our light-sheet microscope, and can be used to analyze neuronal connectivity on the cellular and subcellular level.

The high quality of clearing, which is difficult or impossible to achieve in mature, myelinated brains of adult mice using previously published non-aqueous clearing procedures, was consistently obtained for brains of young and aged mice using the FluoClearBABB method. The improved clearing after denaturation with 1-propanol or *tert*-butanol may be due to their stronger denaturing effect on peptides compared to methanol and ethanol [26,27]. In the case of older mice, it is important to use temperatures slightly higher than room temperature (e.g. 30°C), presumably because this facilitates diffusional exchange and partial denaturation. Comparison of the clearing results of our FluoClearBABB procedure with the original CLARITY protocol is difficult due to the lack of high resolution data provided in ref. [13]. In terms of minimizing optical distortions our clearing procedure appears to be better than another CLARITY-derived protocol [14]. The transparency obtained with our FluoClearBABB protocol seems to be slightly better than with the CUBIC protocol [15].

For the preservation of EGFP fluorescence, both dehydration with water-miscible C3 and C4 alcohols and a basic pH during the clearing steps are essential. However, the reason why C3 and C4 alcohols affect EGFP fluorescence less than the C2 and C1 alcohols in spite of their stronger denaturing effect remains unclear, as does the influence of the pH during clearing in BABB. The pH dependence of EGFP fluorescence in aqueous solution was described before [28].

Fluorescent protein preservation in whole cleared mouse brains has been reported with some aqueous protocols [9,13–15], and has been quantified *in vitro* [15]. While the initial level of fluorescence in these cleared brains appears to be higher than with our FluoClearBABB protocol, long-term stability was either not reported [13–15] or lower [9] than with our protocol.

FluoClearBABB tissue clearing is of high quality and the fluorescence intensity retained is sufficient to permit the analysis of neuronal circuits using high-resolution image stacks obtained from intact brains with light-sheet microscopy. The high resolution achieved throughout the samples shows that the refractive index after clearing is almost uniform, since wave front aberrations would otherwise compromise the resolution. The loss of intensity with depth is moderate, which shows that scattering and absorption have been much reduced by clearing. We used a light-sheet microscope optimized for imaging samples that are prone to shadow formation, but the samples prepared using our method are also suitable for confocal or two-photon microscopy at large depths (data not shown). However, for whole brain imaging the light-sheet approach is much more appropriate since it minimizes out-of-focus photobleaching [6] and provides fast data recordings. The resolution of the LSFM is only marginally less than that of the confocal microscope and both are sufficient for the tracing of axonal projections. Since fluorescence in FluoClearBABB samples was very stable under storage conditions as well as during illumination with light sheet microscopy, we believe that both clearing conditions and the low illumination energy during light-sheet microscopy contribute to fluorescence preservation in the samples.

### Quantitative connectivity analysis in the uncut mouse brain

The FluoClearBABB method—with its high level of fluorescence preservation—allows the spatially resolved detection of small and weakly labelled structures and is thus well suited for the analysis of neuronal connectivity using RABV-based, retrograde transsynaptic labelling. We used a virus mixture of EnvA pseudotyped RABV*ΔG-EGFP*, rAAV- *mRFP1-IRES-TVA* and rAAV-*RG* to retrogradely trace monosynaptic projections onto the EC, and subsequently visualized cells and axons projecting to the virus-injected region. Using this, we could then identify the majority of the expected connections to EC. We are also able to provide the first quantitative comparison between the numbers of monosynaptically connected cells projecting from different individual brain regions onto a spatially defined target cell population in the EC. Beyond the identification and quantitative comparison of projection neurons located in different brain regions, we can—as yet—not draw conclusions about the biological significance of the individually connected brain regions for information processing in the EC. However, the high resolution of the LSFM makes it easy to distinguish cell types based on their morphology. This revealed that a surprisingly large fraction of RABV*∆G-EGFP* positive cells retrogradely labelled in the hippocampus are glia cells, presumably astrocytes labelled by the transfer of RABV*∆G-EGFP* from neurons at synaptiform connections (“tripartite synapses” [29]). The astrocytes were close to EGFP positive fibers, or to fiber bundles that most probably originate from RABV-infected, EGFP positive “source cells” in the EC (see Figs 6E and 6F; 7A; S1 Video). This is a most curious finding, which suggests that our modified RABV has the unique property of jumping selectively from infected neurons to synaptically coupled neurons in a strictly retrograde manner. Interestingly, three recent publications [30–32] using a similar mono-transsynaptic RABV-based tracing system also unexpectedly found labelling of astrocytes after RABV infection of neurons in the hippocampus. All three studies found that the expression of the RABV glycoprotein (RG) in primary infected neurons was absolutely necessary for RABV transport into astrocytes. They suggest that astrocytes form direct physical contacts with RABV-infected neurons. In this context it has also been suggested that astrocytes, as part of a “tripartite” synapse, can provide a stimulatory input to excitatory synapses in the hippocampal dentate gyrus by releasing gliotransmitters onto synaptic boutons via a release apparatus like that in neurons [33].

Thus, we surmise that the EGFP-labelled astrocytes that we have identified in our study form a physical, possibly functional coupling with neurons and are not due to RABV infection resulting from incomplete deletion of the envelope protein. The exact molecular mechanism for this traversal is, however not yet fully understood.

Key benefits of FluoClearBABB are superior clearing performance, long-term stability and photostability of EGFP in clearing solution, and the long-term stability in storage and mechanical rigidity of brains prepared with this protocol. Fast evaluation scans can be acquired without significant loss of fluorescence, followed by high resolution scans (2 hours scan time per hemisphere and channel at an xyz voxel size of 1.6 * 1.6 * 3.2 μm) for the imaging of detailed subcellular cell structures. This has been shown for the round and bushy morphology of glia cells, for the cell shape of pyramidal neurons, interneurons, long-range dendrites and axonal projections up to 5–7 mm in length.

Quantitative analysis of multiple, long-range neuronal connections may take weeks to months to perform, and subsequent re-imaging of selected volumes of interest at high resolution may thus be required. Our method allows specific regions to be revisited by repeated LSFM imaging and at high resolution throughout the brain for at least 8 months, provided the brains are appropriately stored. Long-term stability of the samples also minimizes sample deterioration during the transport required for external collaborations. In addition, the high level of clearing and lack of optical distortions in combination with sample stability will allow future re-evaluation of samples with improved light sheet microscopy [34] and other techniques capable of providing higher resolution images of these samples.

## Conclusion

FluoClearBABB clearing followed by LSFM recording will facilitate detection and analysis of transgenic or virally labelled neurons and neuronal structures such as dendrites and axon bundles throughout the entire brain of both juvenile and adult mice. The long stability in storage of FluoClearBABB-cleared brains allows repeated revisiting of structures at different resolutions and with different light microscopy methods, thus facilitating detailed classification and analysis. Fast data collection from thousands of cells all over the brain, in combination with high-speed algorithms for analysis of possible network differences, may be of great assistance in the detection of impairments in long-range projections in mouse models of neurodegenerative or neurocognitive disorders—with implications for similar conditions in human patients.

## Materials and Methods

### Buffers and solutions

PBS (1.5 mM KH_2_PO_4_ pH7.4, 8 mM Na_2_HPO_4_, 137 mM NaCl, 2.7 mM KCl; PB: 0.1 M NaH_2_PO_4_ pH7.4, 0.1 M Na_2_HPO_4_)_;_ PFA fixative (4% Paraformaldehyde (PFA) dissolved in PBS, pH adjusted to 7.4 with NaOH). Dehydration solutions were prepared from analytical grade alcohols (Sigma) by dilution with double-distilled water. Clearing solution (“BABB”; “Murray’s clear”) was prepared by mixing benzyl alcohol (Merck, analytical grade, P/N 109626) and benzyl benzoate (Sigma, “purissimum p.A.” grade, P/N B6630) in a 1: 2 volume ratio. [4] The pH of dehydration and clearing solutions was adjusted using an InLab Science electrode dedicated for use with organic solvents (P/N 662–2837, Mettler-Toledo) attached to a SevenMulti S40 pH-meter (Mettler-Toledo). Conditioning of the electrode was performed according to [35]. Adjustment of pH was done with triethylamine (Sigma-Aldrich). Sodium ethylate (Sigma-Aldrich) was used for pH adjustment of PB-buffered ethanolic solutions [11]. The pH values of the unadjusted alcoholic and clearing solutions were in the range of 5.0–6.2 (methanol), 6.7–7.4 (ethanol), 6.7–7.9 (1-propanol), 6.3–7.4 (2-Propanol), 5.8–6.7 (*tert*-butanol), and 6.0–6.8 (BABB clearing solution).

### In vitro assay of fluorescent proteins expressed in *E*. *coli*

*E*. *coli* cells (strain BL-21 or XL-1 blue) were transfected with plasmids pET-EGFP (for EGFP expression) or pQE30-MCSTK-mRFP1 (for mRFP1 expression) and grown for at least 24 hours at 37°C. BL-21 cells grown to an O.D_600_ of 0.8 were then treated with 1 mM Isopropyl-β-D-thiogalactopyranoside for another 4 h. *E*. *coli* cells were harvested by pelleting at 8000 g for 2 minutes at 4°C. All subsequent steps were performed at 4°C, if not otherwise stated. Cells were washed once with PBS, resuspended in PBS (same volume as pellet), and fixed by addition of 6 volumes of PFA fixative. After overnight fixation, bacteria were washed three times with PBS, resuspended and stored in one tenth of the initial culture volume of PBS containing 0.05% sodium azide. For the fluorescence assay, an aliquot of the fixed bacteria suspension was pelleted, resuspended in a five-fold volume of melted 1% low-melting agarose (P/N 15510–027, Invitrogen) in PBS, and immediately poured into a glass chamber to generate an agarose gel of 1 mm thickness at RT. Round agarose pieces of 5 mm diameter were punched out and transferred individually into the wells of a 96 well clear-bottom plate (μ-plate, P/N 89626, IBIDI) which proved to be resistant to the organic solvents used. Dehydration was performed stepwise (step cycles 4 h / 4 h / 16 h at RT, if not otherwise indicated) using alcohol / H_2_O mixtures of increasing alcohol concentrations as indicated. Clearing was performed by replacing the dehydration solution with the clearing solution for 4 h and exchanging for fresh clearing solution at the times indicated. Rehydration was performed by first incubating the sample in a mixture of equal volumes of clearing solution and the respective dehydration alcohol, followed by application of the previous alcoholic dehydration steps in reverse sequence (step cycles 4 h / 4 h / 16 h), and at the respective pH, and by three days of incubation in PBS; all steps at RT. Fluorescence readings of individual samples at individual treatment points were performed on a TECAN Spectrafluor multiwell plate fluorescence reader equipped with a High Energy Xenon flashlight bulb for fluorescence excitation. Green fluorescence was recorded with excitation BP 485/20 nm, emission: BP 535/25 nm, and red fluorescence with excitation BP 535/25 nm, emission: BP 595/35 nm. Quantitative analysis of fluorescence was performed with Microsoft Excel 2010 (Microsoft) using macros programmed with Visual Basic for Applications. For each treatment step, average values of quintuplicate positives were corrected for bacterial background by subtraction of the respective averages of triplicate control samples (*E*. *coli* cells without XFP). The resulting values and their standard deviations were plotted as percentages of the respective initial averaged fluorescence value of the samples in PBS.

### Cloning and production of rAAV

For preparation of rAAVs, the RABV-Glycoprotein cDNA from the SAD L16 strain (SAD-G) was subcloned into an rAAV backbone containing a 1.5 kb hybrid chicken beta actin enhancer/CMV promoter (*CAG*), the woodchuck posttranscriptional regulatory element (WPRE), and the bovine growth hormone polyA sequence [36]. The *mRFP1-IRES-TVA* DNA fragment was subcloned into the same rAAV backbone. Recombinant AAV 1/2 pseudotyped viral vectors were generated as described [37]. Briefly, human embryonic kidney 293 (HEK293) cells were transfected with the rAAV plasmid containing the respective cDNAs, and the helper plasmids by standard calcium phosphate transfection. After transfection (48h), the cells were harvested and rAAV were purified using heparin affinity columns. Purification and integrity of the viral capsid proteins (VP1–3) were routinely monitored on a 10% Coomassie-stained SDS/protein gel. The genomic titers were determined using the ABI 7700 real time PCR cycler (Life Technologies) with primers designed to WPRE. Viral titers were ~1 x 10^12^ viral genomes/ml.

### Production of pseudotyped rabies virus

BHK cells were plated on a 15cm Petri dish at a density of ~1.5 x 10^7^. The following day, cells were transfected with 15μg plasmid pCAGG/SAD-G DNA by calcium phosphate transfection. One day later (24h) RABV SAD*ΔG-EGFP* was added at a multiplicity of infection (MOI) of 3. After additional 48h incubation the RABV SAD*ΔG-EGFP* containing supernatant was equally distributed onto four 15cm plates containing pCAGGs/SAD-G (15μg/plate) transfected BHK cells (~1.5 x 10^7^ cells/plate) [20]. Two days later the virus-containing supernatant was applied onto four 15cm plates containing BHK-EnvARGCD cells (~1.5 x 10^7^ cells/plate) at a MOI of 1.5 for pseudotyping. After 12 hours cells were trypsinized and replated onto eight 15cm dishes. Pseudotyped rabies virus-containing supernatant was harvested 2 days later. The supernatant was cleared by 10 minutes centrifugation at 2000 rpm at 4°C and subsequently filtered through a 0.45 μm filter (Nalgene SFCA Bottletop Filter, Thermo Fisher Scientific). The virus of the filtered virus suspension was pelleted by 90 minutes centrifugation at 25000 rpm (SW28, 4°C) in a Beckman 80K ultracentrifuge (Beckman Coulter). After centrifugation the supernatant was discarded. The virus pellet was aspirated in ice-cold PBS and stored in 6μl aliquots at -70°C. Virus titers were determined by serial dilution and overnight infection of primary cortical neurons that had been infected with rAAV1/2 expressing *TVA IRES mCherry* under control of the human synapsin promoter. Three days later the number of fluorescent, SAD*ΔG-EGFP* containing neurons were counted. Titers of RABV SAD*ΔG-EGFP* used for *in vivo* injections were ~2.5 x 10^7^ /ml.

### Animal experiments

Mouse strains used were C57BL/6N, transgenic strain *Tg* ^*CAMKII-PtetlacZ-GFP*^ (G3KT1) carrying a tTA dependent transgene for the humanized EGFP ((hGFP(S65T) [17], expressed under tTA control [38] and strain *Tg* ^*Y5RitTA⁄Y1RVenus*^ expressing neurons in Y5R positive cells [39]. All animal experiments were in compliance with the animal policies of the Max Planck Society and in accordance with the European Community Council Directive of November 24, 1986 (86/609/EEC). Stereotactic viral injections into the brains of anaesthetised mice were approved by the regional council for animal welfare in Karlsruhe (unit 35) under the project license 35.9185.81/G-171/10. The perfusion of the mice was approved by the project license 35.9185.81/G-71/10. Efforts were made to minimize the number of animals used.

### Surgical procedures and virus injections

As approved by governmental, regional council for animal welfare in Karlsruhe (unit 35) under the project licenses 35.9185.81/G-171/10 mice (~10 weeks old) were deeply anaesthetized with a mixture of ketamine and xylazine (100 mg/kg and 10 mg/kg, respectively) injected intraperitoneally, supplemented as necessary whenever the animal showed toe-pinch reflexes. Mice were head-fixed using non-puncture ear bars and a nose clamp (David Kopf Instruments), and their body temperature was maintained at 36–37°C using a rectal probe and a heating blanket (FHC, Bowdoin). An incision was made in the scalp and a small craniotomy was drilled above the relevant area of the lateral entorhinal cortex (LEnt) using a dental drill (Foredom Electric). Subsequently, the dura was removed. The surface of the brain was kept moist by applying sterile PBS (Sigma Aldrich) at regular intervals. For injections, glass pipettes were pulled with a tip size of approximately ~10 μm diameter and tip-filled using negative pressure [40]. Injection pipettes were lowered as accurately as possible. Coordinates for stereotaxic EC injections were: Bregma: -4.48mm, Lateral: 3.5mm, Ventral: 4.4mm. We empirically tested different virus mixtures to achieve optimal retrograde transport. Finally, ~200 nl of virus-containing solution (rAAV and RABV were mixed in a 1: 6 ratio of rAAVs: RABV; rAAV were mixed in a 1: 1 ratio of rAAV1/2 CBA *mRFP1-IRES-TVA*: rAAV1/2 CBA *RG*; genomic titer of rAAVs were determined by RT-PCR and were ~10^12^ genomes/ml; titer of the RABVs were ~2.5 x 10^7^ /ml) was injected under constant positive pressure for ~20 minutes. Following stereotaxic injection, the craniotomy was sealed using a silicone sealant (Kwik-Sil, World Precision Instruments) and the scalp sutured. To minimize the pain for the animal during the recovery phase 20–50 μl Carprofen (50 mg/ml) was injected in the are of the craniotomy before and after the recovery. The time from the initial anaesthesia to completing this procedure was kept as short as possible (~1 hour). During recovery, mice were kept warm on a heating plate (37°C) for ~6 hours.

### Sample fixation and tissue clearing

As approved by governmental, regional council for animal welfare in Karlsruhe (unit 35) under the project licenses 35.9185.81/G-171/10 mice were deeply anaesthetized with a mixture of ketamine and xylazine (100 mg/kg and 10 mg/kg, respectively; Atarost) injected intraperitoneally, and transcardially perfused with an ice-cold solution of 4% paraformaldehyde (PFA) in phosphate buffered saline (PBS). (Virus-injected mice (~10 weeks old) were anesthetized and perfused ten days after the injection of the virus cocktail). Brains were removed and post-fixed at 4°C in 4% PFA for ~20 hours followed by 2 washes in PBS at 4°C. Tissue clearing was performed as described [4], with modifications described in the Results section. Briefly, fixed whole mouse brains stored in PBS were dehydrated, at RT or at 30°C as indicated in the text, by incubation in alcohol / water mixtures of increasing alcohol concentrations (30%, 50%, 70%, 80%, 96%, and twice 100%, each adjusted to the desired pH, or leaving the pH unadjusted), followed twice by clearing solution at the respective pH and temperature for 24 hours. For prolonged storage in clearing medium (> 2 days), samples were stored at 4°C. Imaging was performed at RT at the time points specified, preceded by at least one renewal of the clearing solution.

### Transmission light microscopy

To check clearing on a macroscopic level, mouse brains were placed in glass petri dishes on top of a transillumination light source (3000 K) with a semi-transparent mm scale ruler or a transparent USAF Wheel Target Positive, 1" x 3" (P/N NT59-203, Edmund Optics,) in between light source and dish. Images were taken with a MZ-FL3 stereo binocular microscope (Leica Microsystems) using a D700 SLR camera (Nikon) in transillumination mode (NEF 14bit uncompressed file format) and converted, after white balance of background set to neutral, to TIFF 3 x 16 bit composite format with Photoshop v.5.5 (Adobe Systems) followed by conversion to JPG-RGB and linear contrast adjustment using ImageJ [41]. Note that recording with directed transillumination emphasizes intensity loss caused by scattering and refractive index mismatch. Whole brain linear diameter changes were determined with ImageJ from transmission images including a ruler as an internal reference (rostrocaudal diameter including olfactory bulb and cerebellum, lateral diameter: maximum cortex lateral diameter).

### Light sheet microscopy

#### Setup

A home-built light sheet microscope was designed for fluorescence recording of optically cleared specimens (e.g. whole mouse brain) for both generation of rapid overviews and high resolution z stack mosaic datasets. It covers a wide magnification range (roughly 0.5 x to 50 x) by selected combinations of excitation objectives (focal lengths tested: 50–200 mm), detection objectives (8–200 mm) and detection tube lenses (100–450 mm). In brief, the collimated illumination beam is directed through an arrangement of scanners and lenses to generate a multidirectional, scanned light sheet in the cleared sample. Optical slices are obtained by moving the sample cuvette in z axis, i.e. perpendicular to the light sheet, and recording the fluorescence with a CCD camera. Individual components of our light sheet microscope setup are drawn in Fig 3A: Excitation laser wavelengths were provided by a Coherent Sapphire 488 nm 50 mW or a Coherent Compass 561 nm 50 mW diode-pumped solid state laser (Coherent GmbH). Laser beam merging was performed with silver-coated mirrors (Newport) and dichroic mirrors (Semrock, laser quality, purchased from AHF Analysentechnik) before directing the coaligned laser beams through an acousto-optical tuneable filter (AOTF, type NEOS NA-1500-3-6.5 DEG; NEOS Technologies) which served to control wavelength-specific illumination intensity. A polarization preserving monomode fiber (450–640 nm, manufactured by point source / Qioptiq) with collimation optics on both sides (beam diameters: 0.7 mm) directed the beam into a beam expander (33 x, Keplerian type, assembled from achromat lenses, 6 mm and 200 mm, Linos GmbH) to expand and collimate the laser beam. A motorized iris aperture (OWIS) was used to adjust the diameter of the collimated beam directed to Scanner 1 (typical frequency: 501 Hz, sawtooth waveform, model 6215HSM with 7mm silver-coated mirror, Cambridge Technology Inc.), which was used for light sheet generation (Fig 3A, top left; cf. [19]). Scanner 2 (typical frequency: 52 Hz, sawtooth waveform, model 6240HM with 20mm silver-coated mirror, Cambridge Technology Inc.) generated the multidirectional illumination (Fig 3A, bottom left; [18]). Two additional, achromat type lenses were placed in the scanning system: A scan lens (achromat, f = 100 mm, Linos GmbH) placed after scanner 1, and a tube lens (achromat, f = 150 mm, Linos GmbH) located after scanner 2 (Fig 3A, top left). Illumination objectives used were: Planapo 1x/0.117 (f = 100 mm), or Planapo 2x/0.234 (f = 50 mm), (Fig 3A, left), both of the Leica Macrofluo system. Detection objectives (Fig 3A, right) were either Planapo 0.8x/0.08 (f = 125 mm), Planapo 1x/0.117 (f = 100 mm), or Planapo 2x/0.234 (f = 50 mm) of the Leica Macrofluo system. The 0.8x and the 1x detection objectives were used in combination with the 1x illumination objective, while the 2x detection objective was used together with the 2x illumination objective. Due to refractive index mismatch between clearing solution and air in both the excitation and detection beam path, refocus of both objectives was performed as a function of sample cuvette position. Emission bandpass filters (Semrock, imaging quality, purchased from AHF Analysentechnik) were placed in a Sutter NB10—W32 filter wheel (Fig 3A, right; Sutter Inc.). Detection path tube lens was either a Micro Nikkor 2.8/105 mm (Nikon) or a Micro Nikkor 4/200 mm (Nikon) photographic objective (Fig 3A right), both used at aperture 4 and set to infinity. Linear stages were used for positioning of the sample in x and y direction (type M126.CG1, Physik Instrumente), in z direction and for focusing the excitation and the emission objectives (type M112.2DG, Physik Instrumente). Motorized and electronic components of the system (AOTF, iris aperture, scanners, linear stages, filter wheel and camera) were controlled by software developed with Labview (version 8.6 or version 2011, National Instruments Germany GmbH).

#### Image acquisition

Optically cleared samples were fixed on a needle that was then fixed in place on a silicon cushion located at the bottom of a 20x20x20 mm glass cuvette (2 mm optical glass, Hellma). The cuvette was filled with clearing solution to the top and then covered with a # 1.5 cover slip. To minimize light path length in clearing solution, while avoiding reflections or beam clipping in the excitation path, distances between sample and glass on both the excitation and the emission side were kept at about 1 mm. Image acquisition was controlled by Labview (version 8.6 or version 2011, National Instruments Germany GmbH). EGFP fluorescence was excited at 488 nm, and emission was collected through a bandpass filter (503–537 nm). The red mRFP1 fluorescence was excited by a 561 nm laser, and recorded through a bandpass filter (570–640 nm). The typical laser energy applied per image and mm light sheet width was 4–20 microjoule for each wavelength. Signals were recorded with a cooled black and white CCD camera (Sensicam QE, PCO) at 12 bit grey scale resolution. Typical exposure time was 100 milliseconds per channel. Images were saved to hard disk as OME-TIFF files [42] immediately after acquisition. Metadata log files were generated in ASCII (TXT) and OME-XML format [42].

### Confocal microscopy

Confocal microscopy was performed on a LEICA SP5 confocal scanner on a DM 6000 CFS upright microscope stand equipped with a LEICA HCX APO L20X/0.95 IMM objective dedicated for use with the BABB solution (refractive index: 1.558). Green channel: excitation 488 nm, emission 492–545 nm; red channel: excitation 561 nm, emission 569–628 nm.

### Microscopy data analysis

Microscopy data analysis was performed with using the Fiji distribution (http://pacific.mpi-cbg.de/wiki/index.php/Fiji) of ImageJ. From xyz stacks recorded as mosaic tiles, half brain datasets were generated by stitching the xyz data stacks using an ImageJ plugin (Stitch Viewer, [21], based on a stitcher plugin from [43]). For visualization purposes, correction of the excitation and emission signal intensity falloff caused by brain tissue along the light path was performed with some datasets as follows: First, the location of brain tissue was identified by autofluorescence and FP signal. From an arbitrary, rectangular image background area (>500 x 500 px) outside the brain tissue signal area, a histogram of the stitched original recording was analyzed to determine a threshold for selecting the upper 1% of the intensity values. This threshold was then used to generate a binary 3D mask representing the location of brain tissue. Intensity correction factors were calculated for each voxel within the mask, assuming exponential attenuation of the excitation and emission light in tissue:

$$\text{IntensityCorrection}\;\left(\Delta y,\Delta z\right)=\frac{1}{e^{-k_{y}*\Delta y}*e^{-k_{z}*\Delta z}}$$

where *k*_*y*_ and *k*_*z*_ are the respective absorption coefficients in y and z direction.

This correction factor volume, filtered with a 3D Gaussian (sigma x, y, z: 2, 2, 2), was multiplied with the original 3D dataset to generate the intensity-corrected dataset. Final adjustments of the absorption coefficients were made after visual inspection of the intensity gradient profiles of xy and xz (re-) slices of downscaled datasets.

### Quantitative neuronal connectivity analysis

Monosynaptic connections of RABV*ΔG-EGFP* expressing cells to AAV- *mRFP1-IRES-TVA* / RABV*ΔG-EGFP* labelled cells were analyzed in microscopic 3D datasets by visually marking and saving red and / or green fluorescent cell body locations in 3D space using KNOSSOS software [25]. The preservation of the 3D coordinates of each cell allowed for concomitant assignment to individual clusters representing different brain regions as taken from the Mouse Brain Atlas (Allen Institute for Brain Science). Assignment was double-checked by another investigator. From these numbers, connectivity maps and statistics were generated. Amira software (version 5.5; FEI Corp., Hillsboro) was used to visualize overlays of individually colorized cell clusters with microscopic 3D data and to generate videos. Data were converted with Amira from 16bit greyscale TIFF format to Amira Mesh format. KNOSSOS 3D datasets were converted to Amira Cluster format using code written in MATLAB.

## Supporting Information

S1 Fig Analysis of EGFP fluorescence during dehydration, clearing and rehydration *in vitro* using different conditions. **(A)** Fluorescence of EGFP expressed in *E*. *coli* after dehydration with different alcohols followed by clearing. Dehydration steps were performed as in Fig 1A. All steps were performed at RT and at pH values indicated; Fluorescence was recorded after the second 100% dehydration step (left panel), after 1 and 16 days in clearing solution (center and right panel, respectively); dehydration alcohols are indicated on the x axis: MeOH, methanol; EtOH, ethanol; 1-prop, 1-propanol; 2-prop, 2-propanol; t-but, *tert*-butanol; EtOH/PB: dehydration with increasing concentrations of ethanol in PB at pH 7.4, followed by 100% ethanol (pH unadjusted). Data from same experiment as in Fig 1A. **(B)** EGFP fluorescence after dehydration with *tert*-butanol (each step 1.3 h; first 100% dehydration step: 15 h), followed by 3 days in clearing solution, all steps at RT and at pH values as indicated. **(C)** Preservation of mRFP1 fluorescent protein expressed in *E*. *coli* after 1-propanol or *tert*-butanol dehydration and clearing (each dehydration step lasted 4 h except the incubation at 70% and the second incubation at 100% lasting 16 h each, and additional 5 days in BABB clearing solution; all steps at RT, 30°C, or 37°C as indicated; all solutions at pH 9.5). **(D)** Remaining EGFP fluorescence in *E*. *coli* after dehydration with different alcohols as indicated at the bottom (each step 1.3 h, except for the first 100% dehydration step: 15 h; followed by 3 days in BABB clearing solution). All steps at unadjusted pH (dark grey bars), after raising the pH of the clearing solutions to 9.5 for another 3 days (medium grey bars), and after subsequent rehydration with decreasing concentrations of *tert*-butanol (pH 9.5), followed by three days in PBS (light grey bars), or performing all dehydration and rehydration steps with *tert*-butanol at pH 9.5 (right bar cluster). All experiments were performed at RT.
(TIF)

S2 Fig EGFP fluorescence of olfactory bulbs of a transgenic *Tg* ^*CamKII-EGFP*^ mouse recorded with the light-sheet microscope. Same mouse brain samples as shown in Fig 1. Additional light- sheet microscope recordings after 68 d in clearing solution were added to the panels already shown in Fig 1C. The brightness and contrast settings were set to two different linear ranges (indicated on the left) which were chosen to show the 1P-BABB sample intensity range (upper panels) and the E-BABB sample intensity range (lower panels). gl, glomerular layer; aob, accessory olfactory bulb. Scale bar, 1mm.
(TIF)

S3 Fig Comparison of clearing performance using different protocols. Transmitted light images of whole mouse brains are shown as described for Fig 2. (**A**) Cleared mouse brains from P23 and P67 mice after clearing with original E-BABB procedure or with the 1P-BABB or tB-BABB procedure as indicated. (**B**) Clearing efficiency with P50 mouse brains analyzed after dehydration with pH-unadjusted and pH-adjusted alcohols followed by BABB clearing at the indicated pH. (**C**) Transmitted light images of cleared mouse brains from P50, P142 and P96 mice. Clearing was performed with the E-BABB, 1P-BABB, tB-BABB, EtOH/PB pH 7.4, Clarity (Glycerol clearing), ScaleA2 or the Scale A2/B4/A2 procedure. The two brains from P250 and P673 mice (top right) were cleared using the tB-BABB/30°C procedure. The scaling of all panels is identical. Transparent ruler, short ticks 1 mm. USAF1951 transparent target underneath the brain; Scale bar, 1mm.
(TIF)

S4 Fig Long-term clearing of brains after dehydration with different alcohols / pH values at RT. PFA-fixed whole mouse brains isolated at P44 were dehydrated and cleared as indicated and stored at 4°C 2d after clearing. Transmission images were taken from mouse brains placed on top of a transparent ruler (small ticks, 1 mm) at RT and indicated time points.
(TIF)

S1 Video Long term preservation of fluorescence and sample geometry. Light sheet microscope horizontal recordings of green fluorescence (RABV*ΔG-EGFP*) from a p70 mouse brain were taken at day 32 and day 264, respectively, after onset of clearing. Corresponding subvolumes of the entorhinal cortex / hippocampus region were selected with ImageJ and upscaled to a cubic voxelsize of 1.61 microns with AMIRA software. The day 264 dataset was 3D aligned to the day 32 dataset by affine transformation with AMIRA (Lanczos interpolation). Since the day 32 dataset images had been recorded at three times the excitation power (4.65 microjoule per mm light sheet width) compared with the day 264 dataset images (1.55 microjoule per mm light sheet width), we accounted for this difference by adjusting the image display range with ImageJ accordingly (Set Display Range (32d): 70–1269; (264d): 63–462; with the lower value of each range representing the image background outside the brain tissue signal area). All other recording and image analysis parameters were maintained. Video shows overlapping z maximum projections generated with ImageJ (6.45 microns z projection size each, 3.225 microns z step size increment) of day 32 (**A**), day 264 (**B**) and an overlay of day 32 (red) and day 264 (green) EGFP fluorescence (**C**). Relative z position of the projections is indicated at top right. Bar, 100 microns.
(MP4)

S2 Video Virus injection area. Substack (red/green overlay) at original resolution (voxel size xyz: 1.613 * 1.613 * 3.225 μm) showing a horizontal LSFM recording around the injection site (indicated by a dashed red circle) of a P70 mouse brain. rAAV- *mRFP1-IRES-TVA* cells in red, RABV*ΔG-EGFP* cells in green. Note the strong red autofluorescence of the brain periphery.
(MPG)

S3 Video Coronal maximum projections showing neurons monosynaptically connected to RABV*ΔG-EGFP*(EnvA) infected source cells in EC. Datasets recorded from same brain as for Fig 4, but after 64 days in clearing solution. Horizontal datasets recorded from the right and left hemisphere were corrected for signal attenuation and aligned using the “TransformJ Rotate” plugin of ImageJ (see Materials and Methods section), followed by stitching of both datasets. From coronal re-slices (downsampled to 6.45 μm cubic voxel size), a sequence of maximum projections (25.8 μm z depth each) was generated from both the green and red fluorescence channel. Label (top left) indicates z position of re-slice related to first image. Red arrow indicates virus injection site and direction. Note the strong red autofluorescence at the periphery of the brain.
(MPG)

S4 Video Coronal view showing the position of neurons monosynaptically connected with RABV*ΔG-EGFP*(EnvA) infected source cells. Right hemisphere dataset (same as used for Fig 4) was downsampled to 6.45 μm cubic voxel size and converted to 8 bit Amira Mesh format (color-coded grey). Colored spheres (Amira Cluster format) were overlaid to indicate the 3D position and tissue cell type of EGFP-positive cells taken from the cells’ previous assignment to individual brain regions generated with KNOSSOS software (for color assignment indicated in video, see also Table 2). Abbreviations used: Agran Ins Ctx, Agranular Insular Cortex neurons; Amygd Nuclei, Amygdaloid Nuclei neurons; Ctx, Cortex neurons; DEn, Dorsal Endopiriform Nucleus neurons; DG, Dentate Gyrus Neurons; DG Glia, Dentate Gyrus Glia; Dors Pedunc Cortex, Dorsal Peduncular Cortex neurons; ec, External Capsule neurons; ECT, Ectorhinal Cortex neurons; ec Glia, External Capsule Glia; HC, Hippocampus; HC Alveus Glia, HC Alveus Glia; HC CA1 or, HC CA1 Stratum Oriens neurons; HC CA1 pyr, HC CA1 Stratum Pyramidale neurons; HC CA1 rad, HC CA1 Stratum Radiatum neurons; HC CA3 or, HC CA3 Stratum Oriens neurons; HC CA3 pyr, HC CA3 Stratum Pyramidale neurons; HC CA3 rad, HC CA3 Stratum Radiatum neurons; HDB, Horizontal Diagonal Band neurons; Lat Hypothal Nuclei, Lateral Hypothalamic Nuclei neurons; LEnt, Lateral Entorhinal Cortex neurons; LEnt Glia, Lateral Entorhinal Cortex Glia; MCPO, Magnocellular Preoptic Nucleus neurons; MEnt, Medial Entorhinal Cortex neurons; MEnt Glia, Medial Entorhinal Cortex Glia; MS, Medial Septum; OB, Olfactory Bulb; OB Glomerular Layer, OB Glomerular Layer neurons; OB MC, OB Mitral Cells; Olf Nucleus, Olfactory Nucleus neurons; PiC, Piriform Cortex neurons; PiC Glia, Piriform Cortex Glia; Prh, Perirhinal Cortex neurons; Prh Glia, Perirhinal Cortex Glia; Str Lac Mol, Stratum Lacunosum Moleculare neurons; Str Lac Mol Glia, Stratum Lacunosum Moleculare Glia; Str Glia, Striatum Glia; SUB, Subiculum neurons; Sub Glia, Subiculum Glia; Thal Nuclei, Thalamic Nuclei neurons; VDB, Vertical Diagonal Band neurons; Ventral Orb Ctx, Ventral Orbital Cortex neurons.
(MPG)

S5 Video RABV*ΔG-EGFP*-labeled Glia cells in the mouse brain perforant path. Video animation from a confocal recording of green fluorescence from RABV*ΔG-EGFP* labeled neuronal structures showing neurons and glia cells in close vicinity to source cell axons running along the perforant path. Glia cells show round and bushy morphology, while the neurons are characterized by solid, compact cell bodies with clearly visible neurites. Data were recorded from the same brain hemisphere as shown in Fig 4, after 703 days in FluoClearBABB clearing solution. Voxel size of confocal data set: 0.15 * 0.15 * 0.145 microns, recorded at 12 bit resolution: After deconvolution with Huygens software, the data were converted to 8 bit grayscale for video generation with Amira software.
(MPG)

S6 Video Video animation of RABV*ΔG-EGFP*(EnvA) infected neurons in EC showing axon traces of monosynaptically connected neurons. Right hemisphere horizontal dataset (same as shown in Fig 4) was downsampled to 6.45 μm cubic voxel size and converted from 16 bit greyscale TIFF into 8 bit Amira Mesh format. Four 3D traces of axons manually generated with KNOSSOS software were overlaid as color-coded, solid lines with the EGFP channel (color-coded grey) for generating part of the animation. The trajectory of one of the axons traced with KNOSSOS is depicted by a series of red dots after zooming in to the original 1.61 x 1.61 μm xy pixel size. Note the autofluorescence of the brain periphery.
(MPG)
