# Supplementary figures and images for: Fluorescent-Protein Stabilization and High-Resolution Imaging of Cleared, Intact Mouse Brains
Source: PLoS One. 2015 May 20;10(5):e0124650. doi: 10.1371/journal.pone.0124650 (PMC4439039; doi:10.1371/journal.pone.0124650)

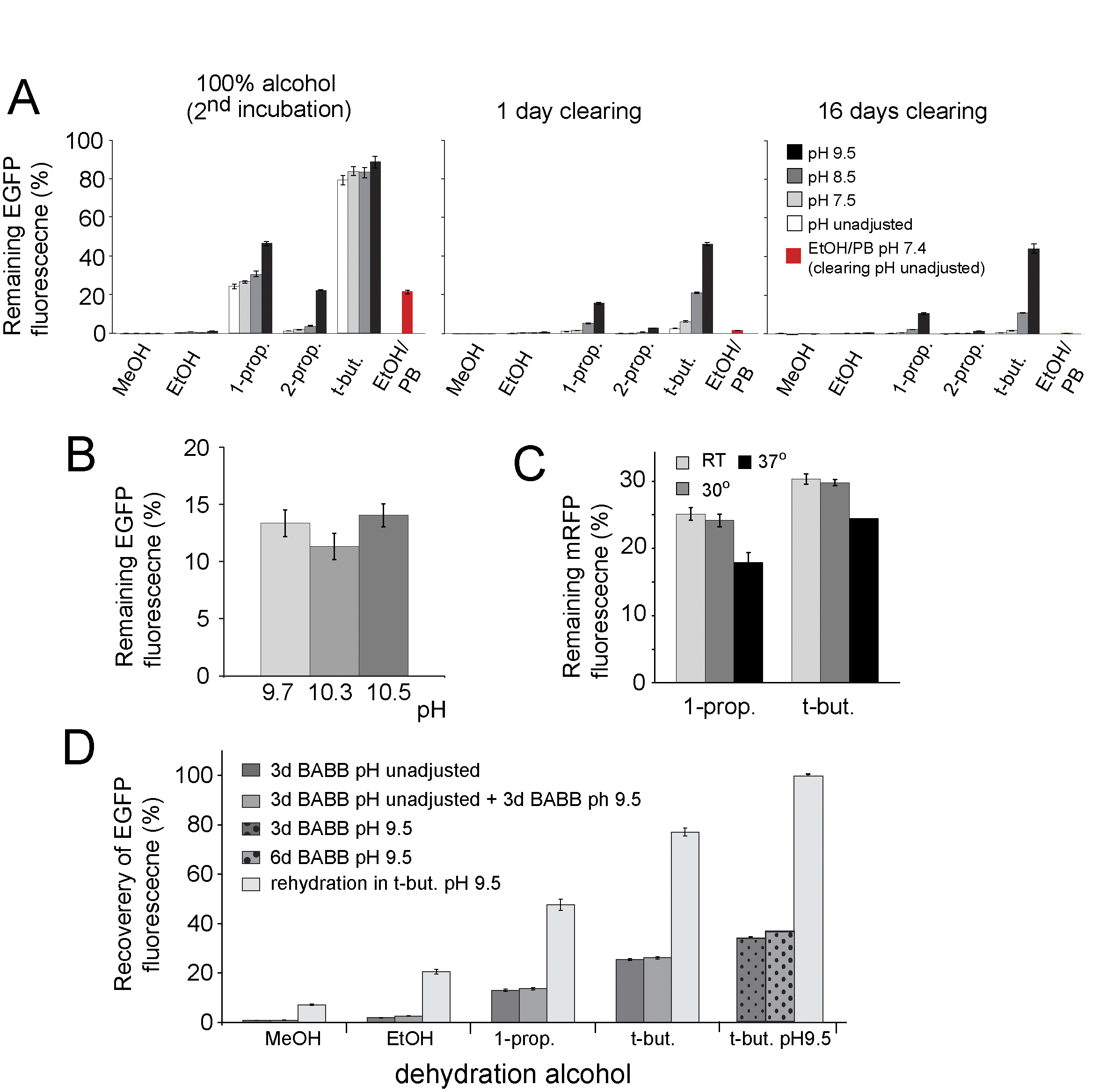

Supplement: S1 Fig — (A) Fluorescence of EGFP expressed in E. coli after dehydration with different alcohols followed by clearing. Dehydration steps were performed as in Fig 1A. All steps were performed at RT and at pH values indicated; Fluorescence was recorded after the second 100% dehydration step (left panel), after 1 and 16 days in clearing solution (center and right panel, respectively); dehydration alcohols are indicated on the x axis: MeOH, methanol; EtOH, ethanol; 1-prop, 1-propanol; 2-prop, 2-propanol; t-but, tert-butanol; EtOH/PB: dehydration with increasing concentrations of ethanol in PB at pH 7.4, followed by 100% ethanol (pH unadjusted). Data from same experiment as in Fig 1A. (B) EGFP fluorescence after dehydration with tert-butanol (each step 1.3 h; first 100% dehydration step: 15 h), followed by 3 days in clearing solution, all steps at RT and at pH values as indicated. (C) Preservation of mRFP1 fluorescent protein expressed in E. coli after 1-propanol or tert-butanol dehydration and clearing (each dehydration step lasted 4 h except the incubation at 70% and the second incubation at 100% lasting 16 h each, and additional 5 days in BABB clearing solution; all steps at RT, 30°C, or 37°C as indicated; all solutions at pH 9.5). (D) Remaining EGFP fluorescence in E. coli after dehydration with different alcohols as indicated at the bottom (each step 1.3 h, except for the first 100% dehydration step: 15 h; followed by 3 days in BABB clearing solution). All steps at unadjusted pH (dark grey bars), after raising the pH of the clearing solutions to 9.5 for another 3 days (medium grey bars), and after subsequent rehydration with decreasing concentrations of tert-butanol (pH 9.5), followed by three days in PBS (light grey bars), or performing all dehydration and rehydration steps with tert-butanol at pH 9.5 (right bar cluster). All experiments were performed at RT. (TIF) [file pone.0124650.s001.tif]

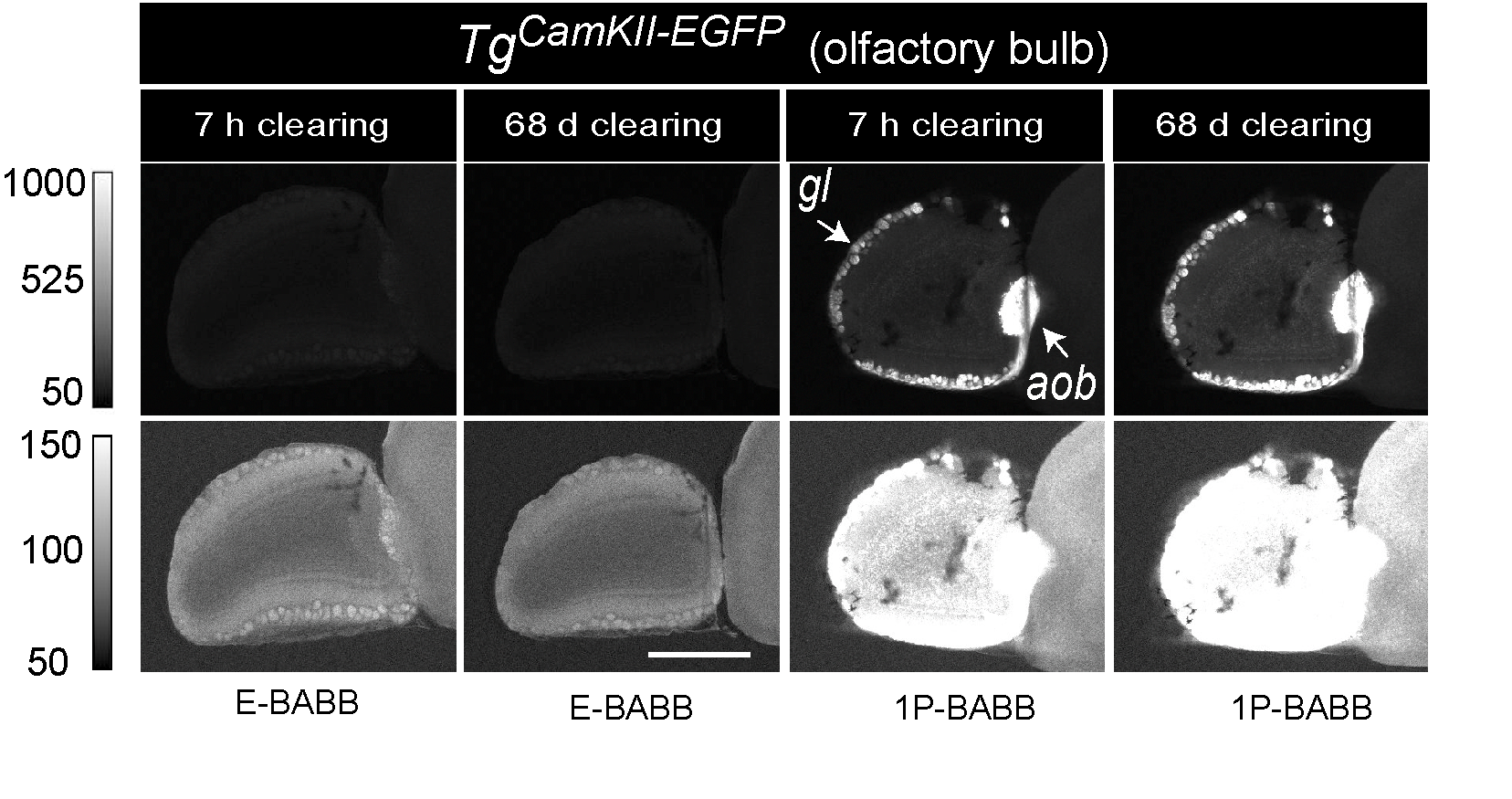

Supplement: S2 Fig — Same mouse brain samples as shown in Fig 1. Additional light- sheet microscope recordings after 68 d in clearing solution were added to the panels already shown in Fig 1C. The brightness and contrast settings were set to two different linear ranges (indicated on the left) which were chosen to show the 1P-BABB sample intensity range (upper panels) and the E-BABB sample intensity range (lower panels). gl, glomerular layer; aob, accessory olfactory bulb. Scale bar, 1mm. (TIF) [file pone.0124650.s002.tif]

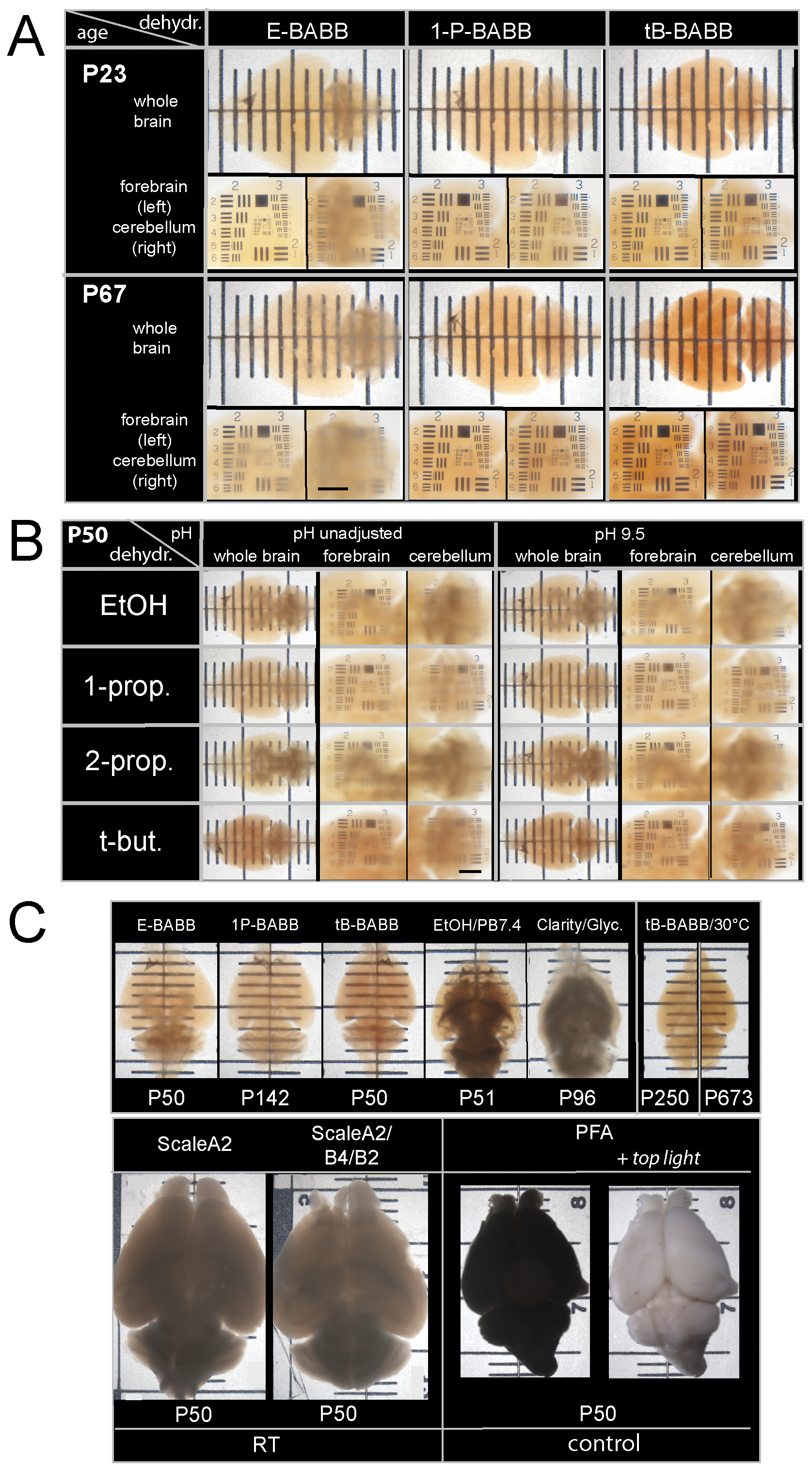

Supplement: S3 Fig — Transmitted light images of whole mouse brains are shown as described for Fig 2. (A) Cleared mouse brains from P23 and P67 mice after clearing with original E-BABB procedure or with the 1P-BABB or tB-BABB procedure as indicated. (B) Clearing efficiency with P50 mouse brains analyzed after dehydration with pH-unadjusted and pH-adjusted alcohols followed by BABB clearing at the indicated pH. (C) Transmitted light images of cleared mouse brains from P50, P142 and P96 mice. Clearing was performed with the E-BABB, 1P-BABB, tB-BABB, EtOH/PB pH 7.4, Clarity (Glycerol clearing), ScaleA2 or the Scale A2/B4/A2 procedure. The two brains from P250 and P673 mice (top right) were cleared using the tB-BABB/30°C procedure. The scaling of all panels is identical. Transparent ruler, short ticks 1 mm. USAF1951 transparent target underneath the brain; Scale bar, 1mm. (TIF) [file pone.0124650.s003.tif]

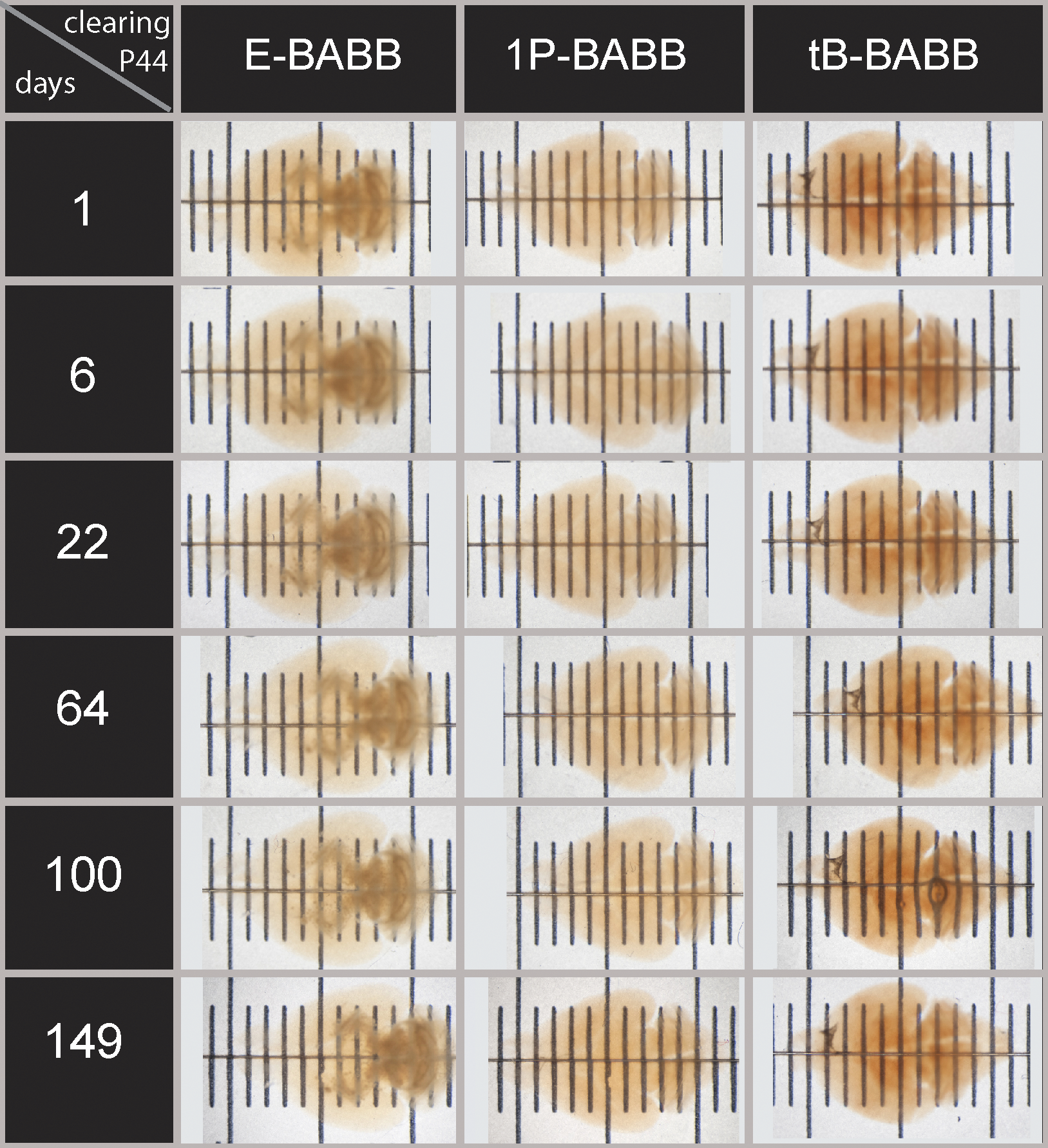

Supplement: S4 Fig — PFA-fixed whole mouse brains isolated at P44 were dehydrated and cleared as indicated and stored at 4°C 2d after clearing. Transmission images were taken from mouse brains placed on top of a transparent ruler (small ticks, 1 mm) at RT and indicated time points. (TIF) [file pone.0124650.s004.tif]
